# Carfilzomib Enhances the Suppressive Effect of Ruxolitinib in Myelofibrosis

**DOI:** 10.3390/cancers13194863

**Published:** 2021-09-28

**Authors:** Simone Claudiani, Clinton C. Mason, Dragana Milojkovic, Andrea Bianchi, Cristina Pellegrini, Antinisca Di Marco, Carme R. Fiol, Mark Robinson, Kanagaraju Ponnusamy, Katya Mokretar, Avirup Chowdhury, Michael Albert, Alistair G. Reid, Michael W. Deininger, Kikkeri Naresh, Jane F. Apperley, Jamshid S. Khorashad

**Affiliations:** 1 Centre for Haematology, Department of Immunology and Inflammation, Imperial College, London W12 0NN, UK; s.claudiani@imperial.ac.uk (S.C.); d.milojkovic@imperial.ac.uk (D.M.); carme.fiol.19@ucl.ac.uk (C.R.F.); marrobinson@coh.org (M.R.); k.ponnusamy@imperial.ac.uk (K.P.); katya.mokretar@viapath.org (K.M.); avirupchowdhury@nhs.net (A.C.); michaelalbert2020@u.northwestern.edu (M.A.); knaresh@fredhutch.org (K.N.); j.apperley@imperial.ac.uk (J.F.A.); 2 Department of Pediatrics, Division of Pediatric Hematology and Oncology, University of Utah, Salt Lake City, UT 84108, USA; clint.mason@hci.utah.edu; 3 Department of Information Engineering, University of L’Aquila, 67100 L’Aquila, Italy; andrea.bianchi@graduate.univaq.it (A.B.); antinisca.dimarco@univaq.it (A.D.M.); 4 Department of Biotechnological and Applied Clinical Science, University of L’Aquila, 67100 L’Aquila, Italy; cristina.pellegrini@univaq.it; 5 Molecular Pathology Unit, Liverpool University, Liverpool L7 8XP, UK; alistair.reid1@nhs.net; 6 Versiti Blood Research Institute, Division of Hematology and Oncology, Department of Medicine, Medical College of Wisconsin, Milwaukee, WI 53226, USA; michael.deininger@hci.utah.edu

**Keywords:** myelofibrosis, ruxolitinib, carfilzomib, shRNA library screen

## Abstract

**Simple Summary:**

Myelofibrosis (MF) is a progressive myeloproliferative neoplasm with tendency towards leukemic transformation and has the poorest prognosis amongst the Philadelphia-negative classical myeloproliferative neoplasms (MPN). Ruxolitinib, the first FDA-approved JAK1/2 tyrosine kinase inhibitor, is efficient in reducing spleen size and improving patient symptoms, but it has not been shown to eradicate the MF clone. In this study, using functional genomics techniques, we identified the proteasome family as an additional therapeutic target and demonstrated that inhibition of the proteasome family by carfilzomib in combination with ruxolitinib enhanced suppression of primary MF cells in vitro.

**Abstract:**

As the first FDA-approved tyrosine kinase inhibitor for treatment of patients with myelofibrosis (MF), ruxolitinib improves clinical symptoms but does not lead to eradication of the disease or significant reduction of the mutated allele burden. The resistance of MF clones against the suppressive action of ruxolitinib may be due to intrinsic or extrinsic mechanisms leading to activity of additional pro-survival genes or signalling pathways that function independently of JAK2/STAT5. To identify alternative therapeutic targets, we applied a pooled-shRNA library targeting ~5000 genes to a *JAK2*^V617F^-positive cell line under a variety of conditions, including absence or presence of ruxolitinib and in the presence of a bone marrow microenvironment-like culture medium. We identified several proteasomal gene family members as essential to HEL cell survival. The importance of these genes was validated in MF cells using the proteasomal inhibitor carfilzomib, which also enhanced lethality in combination with ruxolitinib. We also showed that proteasome gene expression is reduced by ruxolitinib in MF CD34^+^ cells and that additional targeting of proteasomal activity by carfilzomib enhances the inhibitory action of ruxolitinib in vitro. Hence, this study suggests a potential role for proteasome inhibitors in combination with ruxolitinib for management of MF patients.

## 1. Introduction

Philadelphia-negative classical myeloproliferative neoplasms (MPN), comprising essential thrombocythemia (ET), polycythaemia vera (PV) and myelofibrosis (MF) are clonal disorders of the haematopoietic stem cell, characterised by abnormal proliferation of the myeloid compartment with maintained differentiation, decreased apoptosis and defective maturation of megakaryocytes. These conditions, in a minority of cases, can evolve into acute myeloid leukaemia [1]. MF is a progressive MPN characterized by extramedullary haematopoiesis, bone marrow fibrosis, and a tendency towards leukemic transformation and has the poorest prognosis [2,3].

Mutations in the genes *JAK2*, *CALR*, and *MPL* are well-known drivers of MPN. Discovery of these mutations and demonstration of their association with upregulation of *JAK*/*STAT* signalling paved the way to the development of new therapy. Ruxolitinib is an FDA approved *JAK1*/*2* inhibitor for the management of MF [4]. Two clinical trials evaluating ruxolitinib in MF (COMFORT I and II) [5,6] showed efficacy in reducing spleen size and improving patient symptoms, with a marginal improvement in survival [7], but did not show a significant change in the natural history of the disease [8,9] nor eradication of the MF clone. This may be due to activation of alternative intrinsic or extrinsic pathways which provide an additional survival mechanism for the cells when the *JAK2* pathway is inhibited. The activity of *YBX1* in the maintenance of *JAK2*^V617F^ malignant clones in the presence of *JAK2* inhibitors is an example of a cell-intrinsic mechanism [10]. An extrinsic mechanism may activate parallel viable signals through physical contact with the cells and matrix of the microenvironment or secretion of cytokines from cellular components, such as mesenchymal stromal cells [11,12].

To identify genes contributing to the survival of ruxolitinib-treated MF cells, we applied a large pooled-shRNA library to a *JAK2*^V617F^-positive cell line in different settings and also performed RNA sequencing on primary MF cells exposed to ruxolitinib. We found that the activity of the proteasome has an essential role for the viability of MF cells.

## 2. Materials and Methods

### 2.1. Samples

Peripheral blood samples were obtained from MF patients who provided consent to the use of their blood for research purposes. Patient clinical characteristics are summarised in Supplementary Table S1. CD34^+^ cells were positively selected from mononuclear cells (MNC) using CD34^+^MicroBead kit (Miltenyi Biotec, Bergisch Gladbach, Germany) as recommended by the manufacturer. CD34^+^ cells were cultured in StemSpan medium supplemented with CC-100 human cytokine cocktail (Stem Cell Technologies, Vancouver, Canada) for 48 hours (h) before use.

### 2.2. Cell Line Preparation and Measurement of Sensitivity to Ruxolitinib

The human erythroleukemia cell line (HEL), with homozygous expression of *JAK2*^V617F^ (Leibniz Institute DSMZ, Braunschweig, Germany) was grown in RF10 (RPMI media with 10% FBS, 1% penicillin/streptomycin and 1% L-glutamine, Sigma-Aldrich, St. Louis, MO, USA), as previously described [13,14,15]. The human marrow stromal cell line, HS-5 (ATCC, Manassas, VA, USA) was cultured in RF10. Conditioned media (CM) was prepared as described previously [16], filtered, and stored at −80 °C. Puromycin resistance was achieved by transformation with a pGIPZ construct (Addgene, Watertown, MA, USA) carrying a puromycin resistance gene. HEL cells were seeded on a 6-well plate and cultured with different concentrations of ruxolitinib (0, 150, 300, 500, 750 and 1000 nM) in RF10 or HS-5 CM [16]. The proliferation assay was performed through 3-(4,5-dimethylthiazol-2-yl)-5-(3-carboxymethoxyphenyl)-2-(4-sulfophenyl)-2H-tetrazolium inner salt (MTS; Promega, Madison, WI, USA) (MTS) assay, as described previously [17].

Apoptosis was assessed by staining the primary cells with FITC-conjugated Annexin V in combination with propidium iodide as recommended by the manufacturer (Miltenyi Biotec, Bergisch Gladbach, Germany) before flow–cytometric analysis.

### 2.3. Pooled-shRNA Library Screen

**Figure 1:**
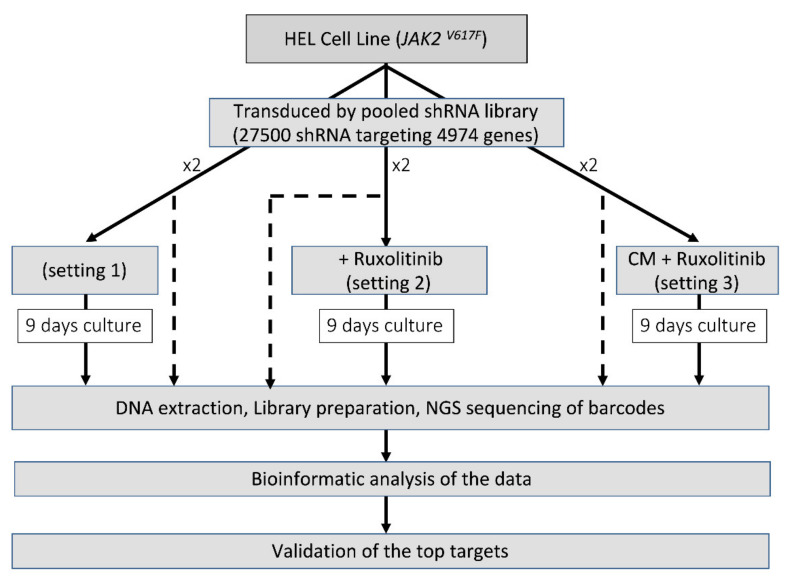
Identification of the essential survival genes in different settings. For each setting, before the selection period in culture, one third of the cells were stored to represent the shRNA frequency in the transduced cells (shown as dash).

We screened the HEL cells under various conditions using the Decipher Human Module 1 shRNA library (Cellecta, Mountain View, CA, USA) which comprises 5043 targets of major intracellular signalling pathways (corresponding to 4974 reliable genes) with 27,500 shRNA (26 937 shRNAs targeting the reliable genes). We optimised the methodology such that the final number of infected cells would be at least 200 to 1000 times the complexity of the shRNA library. Each experiment was performed in duplicate from the transduction stage forward, and hence the replicates reflect not only technical sequencing variation but also variation in the entire shRNA integration process. Ruxolitinib has previously been shown to have a strong effect in reducing the phosphorylation of *JAK2*, *STAT3* and *STAT5* within 2–4 h from exposure, at a concentration of 100 nM [18,19]. We chose the low inhibitory dose of 150 nM for the 9-day selection period of the pooled-shRNA library screen to allow for inhibition of *JAK2*/*STAT5* phosphorylation without a significant reduction in the number of cells, allowing the opportunity for cells to activate alternative protective pathways. Cell transduction was performed as described previously [17]. At 72 h, cells were analysed for red fluorescent protein (RFP) expression (the fluorescent marker of the shRNA construct) and experiments with a transduction efficiency of >40% and <10% were excluded. One third of the cells were snap-frozen for use as control and the rest were cultured for 9 days in the presence of 2 µg/µL puromycin to enrich the transduced cells in three different settings: (1) RF10 medium (2) RF10 + ruxolitinib (150 nM); (3) HS-5 CM only + ruxolitinib (added daily at a concentration of 150 nM) for the first 3 days, followed by the combination of HS-5 CM (50% of the volume) and RF10 (50% of the volume) + ruxolitinib. The combination of CM with RF10 for the last 6 days of culture in setting 3 was to avoid the depletion of the medium from metabolic components. Cells were collected at day 9 [17] (Figure 1).

### 2.4. DNA Extraction, shRNA, Bioinformatics and Statistical Analyses

DNA was extracted from cell pellets collected at baseline, and after the selection period, used for amplification of the 27,500 shRNA barcodes using LNGS-101, NGS Prep Kit for shRNA libraries in pRSI12 and purified according to manufacturer recommendations. Samples were randomized prior to sequencing on a NextSeq 500 Sequencing System (Illumina, San Diego, CA, USA) to reduce potential biases associated with variable sequencing depths and other NGS-related artefacts [20]. Bioinformatic analyses and fold change calculations were performed as previously described [17,21]. In brief, the barcodes for each shRNA were identified in the sequencing data for each sample. Tallying and subsequent normalization of shRNA read counts allowed for fold change calculations to assess the degree of depletion of each shRNA in surviving cells from those incorporated into the cells at baseline. Importantly, samples were sequenced and reads compared between both baseline and follow-up to control for the possibility of fold change bias which can be caused by dissimilar shRNA incorporation from levels provided in a surrogate baseline file provided by the manufacturer for experiments which omit baseline assessment. Statistical analyses of the data were performed in SAS (version 9.1). Figures were generated with Prism GraphPad (version 8.4).

### 2.5. Chemical Validation of Targets

Chemical validation of targets identified in the pooled-shRNA screen was conducted on both the HEL cell line and primary MF cells. For the MTS assay, 3–5 × 10^3^ HEL cells were seeded in 100 µL of RF10 in each well of a 96-well plate and cultured for 72 h in the presence of ruxolitinib 300 nM ± carfilzomib (CFZ) at 10 nM or 50 nM. For proteasome inhibitor IC_50_ determination, HEL cells were exposed to increasing concentrations (0–80 nM) of bortezomib or CFZ for 72 h. Experiments contained 3–5 technical replicates. Potential additive or synergistic effects of drug combination were investigated using the Chou-Talalay method [22], by means of CompuSyn software (ComboSyn, Inc., Paramus, NJ, USA).

Cell viability on primary cells was assessed by trypan blue exclusion at different time points and cells were counted with an automated cell counter (Countess II FL, Thermo Fisher Scientific, Waltham, MA, USA). For MTS assay on primary cells (CD34^+^ or MNC), 2–5 × 10^4^ cells were suspended in 100 µL StemSpan medium in the presence of inhibitors in each well of a 96-well plate, with at least triplicate assessment. For details, please refer to Supplementary Materials and Methods.

### 2.6. Colony Formation Assay

CD34^+^ cells from MF patients were exposed to inhibitors in two different settings: continuous or pulsed drug exposure. Under continuous exposure, cells were plated with ruxolitinib and CFZ. For the pulsed exposure, after 1-h incubation with CFZ, cells were washed and plated in the presence of ruxolitinib (Figure S3). The cell plating in methylcellulose was performed as described previously [17]. Each experiment was run in duplicate, and colonies were manually counted after 14 days.

### 2.7. RNA Sequencing and Analysis

Detailed information on RNA-seq experiments is provided in the Supplementary Materials and Methods. RNA-seq data were generated from CD34^+^ cells of 3 MF patients (patients MF#3, MF#8 and MF#10, listed in Table S1) after 48 h of in vitro treatment with 300 nM ruxolitinib or in untreated cells for each sample. Sequencing was performed on a NextSeq 500 (Illumina, San Diego, CA, USA). The RNA-seq data were mapped to the GRCh38 human genomic reference DNA sequence (https://www.ensembl.org/Homo_sapiens/Info/Index, accessed on 11 June 2020) using STAR (https://github.com/alexdobin/STAR version ‘2.7.5a’, accessed on 11 June 2020).

The tallying of read counts mapping to each gene was performed using the ‘featureCounts’ program (http://subread.sourceforge.net (version ‘2.0.1’)). The reads were normalised to the control samples and the differential expression analysis was performed using the ‘DESeq2’ package (version ‘1.26.0’). Heat maps were produced using the ‘pheatmap’ package in R (Version 3.6.0) and included only genes showing padj and *p* value < 0.05 and log_2_ fold change > 1 in absolute value. The Gene Set Enrichment Analysis was performed with the ‘GSEA’ software version ‘4.0.3’ (https://www.gsea-msigdb.org/gsea/index.jsp), by using canonical pathways of KEGG and BIOCARTA as the reference database (version ‘7.1’).

## 3. Results

### 3.1. Pooled-shRNA Library Screen Identified Proteasome Family Activity Essential for the Viability of the HEL Cells in the Presence or Absence of Ruxolitinib

We performed a large, pooled-shRNA library screen to identify genes essential for the viability of HEL cells through negative selection. Cells harbouring an shRNA which targeted an essential gene for viability would be depleted (negatively enriched compared to baseline, evaluated as a fold-change reduction) following 9 days of culture (selection period). In vitro treatment of HEL cells with increasing doses of ruxolitinib (0, 150, 300, 500, 750 and 1000 nM) demonstrated reduced sensitivity to ruxolitinib in cells cultured in HS-5 derived CM, compared to non-CM, suggesting a protective role of the microenvironment against ruxolitinib-induced inhibition (Figure S1), and supporting the notion that modelling the microenvironment is an important consideration for in vitro investigations of drug resistance. The ruxolitinib IC_50_ for HEL cells was 1299 nM, which increased to 3412 nM in the presence of HS-5 medium (Figure S1). We applied a pooled-shRNA library screen to identify the mechanisms of resistance to ruxolitinib as we had previously described in chronic myeloid leukaemia [17], with modifications. Identification of essential genes for the survival of HEL cells in the presence or absence of ruxolitinib was performed in three settings (Figure 1). In the first setting, we identified the top essential genes for the survival of HEL cells in the absence of ruxolitinib. These genes were used as a basis for distinguishing emerging survival genes in HEL cells treated with ruxolitinib with and without protective microenvironment-mimicking factors from HS-5-CM (settings 2 and 3, respectively).

NGS of the shRNA barcodes showed the recovery of nearly all 27,500 unique barcodes in each experiment, consistent with complete representation of the shRNAs in the library. The reproducibility of the screen was assessed by comparing entire experimental replicates (in contrast to only technical sequencing replicates) for the fold change depletions of the 26,937 shRNAs observed (Figure S2). The fold changes in barcode abundance between baseline and the time periods for selection were highly correlated for each setting with many of the highest-ranking genes retaining their high rank, confirming the reproducibility of this technique.

The genes essential for HEL cell survival were identified using a bioinformatic pipeline we have previously described [17]. Genes were ranked based on the second largest fold change among the shRNA targeting each of the 4974 genes (each gene was targeted with either five or six distinct shRNAs). Hence, for each ranked shRNA, there was another shRNA for the same gene with a larger fold change between baseline and follow-up. The average number of total reads per sample was 24.7 million (range: 19.1 to 34.8 million).

**Figure 2:**
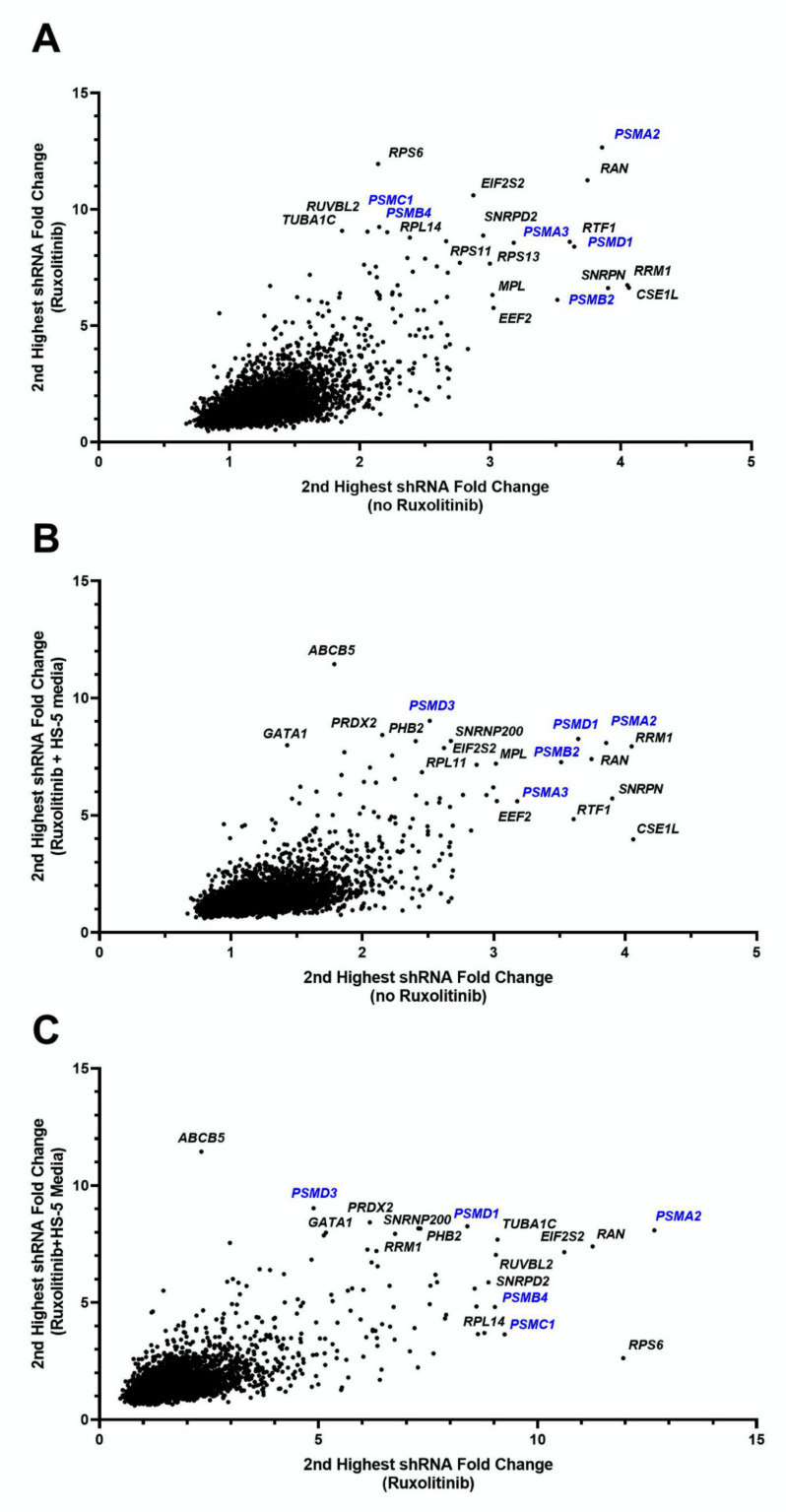
Fold change of shRNAs for HEL cell line under various conditions. Each figure shows the second highest fold change for shRNAs targeting each of 4974 genes, showing comparisons for each combination of conditions. Genes in the uppermost right corners indicate genes which under both of the compared conditions had high shRNA depletion at follow-up compared to baseline, indicating essentiality of the gene for HEL cells survival. (**A**) Fold change of ruxolitinib treated cells to baseline (vertical axis) versus cells cultured without ruxolitinib to baseline (horizontal axis). (**B**) Fold change of HS-5 media plus ruxolitinib treated cells to baseline (vertical axis) versus cells cultured without ruxolitinib to baseline (horizontal axis). (**C**) Fold change of HS-5 media plus ruxolitinib treated cells to baseline (vertical axis) versus cells cultured with ruxolitinib (and no HS-5 media) to baseline (horizontal axis). The first replicate (run 1) values are shown for each setting. These data indicate a significant abundance of proteasome genes (in blue font), requisite for the survival of HEL cells regardless of microenvironment or exposure to ruxolitinib.

For one of the experimental replicates, 7 of the top 25 genes essential for the viability of HEL cells (setting 1) belonged to the proteasomal family with three of these ranking as the most essential genes in the list (*PSMD1*, *PSMB2*, *PSMA2*, Figure 2, Table S2). Screening of HEL cells in the presence of 150 nM ruxolitinib (setting 2) reproduced the majority of the essential genes that had been observed in the absence of ruxolitinib (Table S2).

To investigate the potential role of the microenvironment on the selection of genes with a pro-survival function in the presence of ruxolitinib, we performed the pooled-shRNA library screen on HEL cells treated with ruxolitinib and cultured in HS-5 CM (setting 3). In this setting, nucleo-cytoplasmic transport, proteasome machinery, and several other genes that had ranked highly in settings 1 and 2 maintained their importance. Three genes in the top 1% of essential genes for both replicate experiments of setting 3 had not been present within the top 1% for any of the experiments performed for settings 1 and 2, one of which was a member of the proteasomal family (*PSMB1*, *SNRPA1*, *SIN3A*) (Table S2).

Given the significance of the proteasomal gene family for the survival of HEL cells irrespective of ruxolitinib exposure or microenvironment mimicking factors, and recent data on the higher expression of proteasomal genes in MF patients [23,24] we further explored the therapeutic effect of proteasome inhibition in combination with ruxolitinib.

### 3.2. HEL Cells and Primary MF Cells Demonstrated High Sensitivity to Combined Inhibition of Proteasomes and JAK2/STAT5

**Figure 3:**
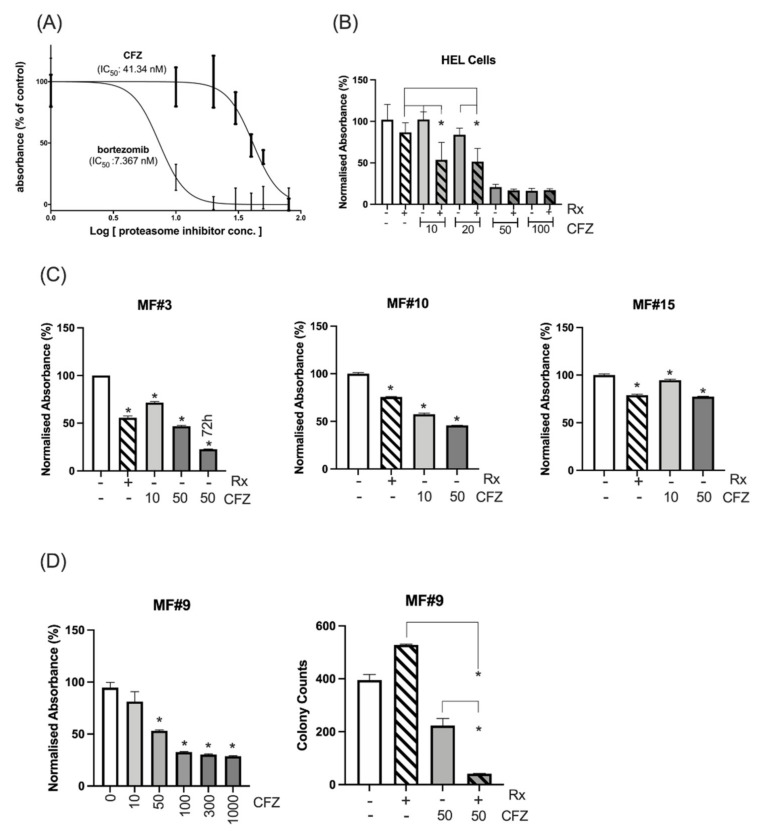
Cell line and primary MF cells’ sensitivity to proteasome inhibitors. (**A**) The IC_50_ was measured for CFZ and bortezomib after measuring the proliferation and viability at 72 h using MTS assay. (**B**) Sensitivity of HEL cells to various doses of CFZ alone or in combination with 300 nM ruxolitinib (Rx). Combination of ruxolitinib with CFZ at 10 or 20 nM significantly increased the inhibition of the HEL cells compared to either ruxolitinib (*p* value: 0.01 and 0.005 respectively) or CFZ (*p* value: 0.001 or 0.01 respectively) after 72 h. * represents *p* value < 0.05 for the comparison between the combination therapy and either treatment. The inhibition at 50 or 100 nM CFZ alone or in combination compared to control was significant. The *Y* axis represents the normalized absorbance to untreated control. (**C**) CD34^+^ cells from 3 MF patients showed sensitivity to 10 and 50 nM CFZ as measured by MTS assay at 24 h (For MF#3 the viability of the cells at 50 nM CFZ was also measured at 72 h). Y axis represents the % of absorbance relative to the untreated control. The columns represent the mean from at least 3 technical replicates (* represents a significant *p* value in comparison to untreated control). Please see the *p* values of the comparison between any two conditions in Table S3. (**D**) (Left) Ruxolitinib resistant triple-negative MF#9 showed sensitivity to CFZ, and this sensitivity became significant at 50 nM (*p* = 0.0001) with higher doses of CFZ leading to higher suppression as measured by MTS assay at 48 h (results are presented as mean and error bars of at least 3 replicates). The Y axis represents the normalised absorbance of the MTS assay. (Right) Colony formation assay shows CFZ has a significant inhibitory impact on stem/progenitor cells at 50 nM which increased several times in combination with ruxolitinib. The data for each condition was achieved by reading the number of colonies from two dishes (duplicates for each condition) and presented with error bars (*: *p* value < 0.05).

Bortezomib and CFZ are clinically approved proteasome inhibitors. IC_50_ values for CFZ and bortezomib in the HEL cells were 41.3 and 7.3 nM respectively (Figure 3A). Due to higher efficacy of CFZ over bortezomib in multiple myeloma [25,26,27] and its better clinical toxicity profile, we continued the validation using CFZ. HEL cells were treated with various doses of CFZ around the IC_50_ as both a single agent and in combination with 300 nM ruxolitinib to investigate potential synergism between the two inhibitors. A combination of the two agents led to significant reduction of viability and proliferation using 10 nM and 20 nM CFZ (Figure 3B). The combination index for the two latter CFZ concentrations with ruxolitinib 300 nM were 0.56 and 0.78, respectively, in keeping with synergism and moderate synergism, respectively. The combination was not significant at 50 or 100 nM compared to CFZ alone, perhaps because the suppressive effect of CFZ at this dose did not leave any room for additional contribution from ruxolitinib. These data validated our finding from the pooled-shRNA library regarding the significance of the proteasomal machinery for viability and proliferation of the MPN cell line model.

Next, the proteasome machinery was investigated as a target in CD34^+^ cells from MF patients. We used 300 nM ruxolitinib which is a value between the ruxolitinib C_max_ of 1100 nM after a single oral dose of 25 mg and the concentration of 45.6 nM after 12 h (when the next dose is given) [28]. The in vitro treatment of CD34^+^ cells from three patients (MF#3, #10, #15) showed that CFZ at 10 or 50 nM had a significant inhibitory effect on MF cells at 24 h and the inhibition further increased at 72 h, as shown for MF#3 (Figure 3C). To observe whether CFZ is effective in a triple-negative ruxolitinib-resistant MF patient, CD34^+^ cells from patient MF#9, who had shown progressive splenomegaly associated with lack of symptom control and no improvement of anaemia, were treated with CFZ at various doses ranging from 10 to 1000 nM and their viability/proliferation was measured at 48 h using MTS assay. CD34^+^ cells from this resistant patient showed a dose-associated sensitivity to CFZ (Figure 3D, left). The colony counts at day 14 did not show any inhibitory effect on colony formation by ruxolitinib but CFZ at 50 nM significantly reduced the number of colonies and demonstrated further reduction in combination with ruxolitinib (Figure 3D, right). These data suggest that CFZ may enhance the suppressive action of ruxolitinib, when ruxolitinib as a single agent has no inhibitory effect.

**Figure 4:**
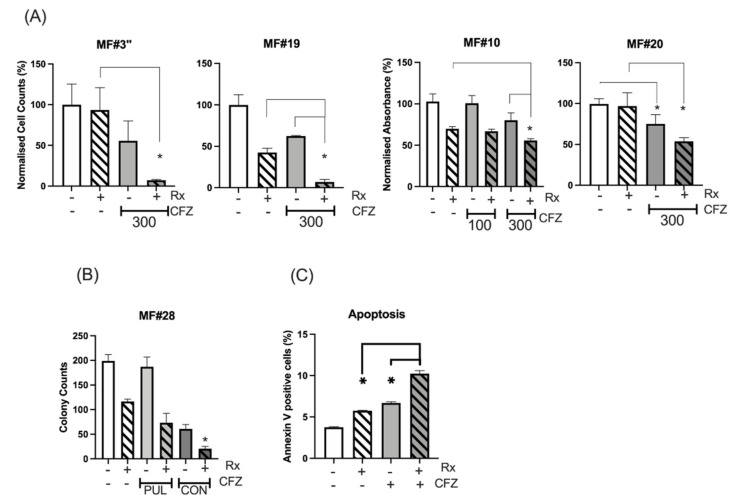
Continuous and pulsed exposure of MF CD34^+^ cells to CFZ. (**A**) CFZ 1h-pulsed exposure potentiated the ruxolitinib effect on the inhibition of cell proliferation or viability of MNC from MF#3”, MF#19 and MF#20 and CD34^+^ cells from MF#10. The cell count (MF#3” and MF#19) and the MTS assay measurement (MF#10 and MF#20) was performed at 72 h (MF19, MF#20 and MF#10) and at 48 h (MF#3) from pulsed exposure (refer to Figure S3 for experiment description). The *Y* axis represents the normalised cell counts (MF#3 and MF#19) or normalised absorbance (MF#10 and MF#20) expressed as percentage of the control. Cell viability was significantly reduced with 300 nM 1h-pulsed CFZ compared to untreated control (*p* value < 0.05). The reduction of cell viability by ruxolitinib was also significant (*p* value < 0.05) except for MF#3” and MF#20 (* represents *p* value < 0.05). Please see the *p* values of the comparison between any two conditions in Table S4. (**B**) The colony formation assay was set up for CD34^+^ cells (MF#28) after 24 h of continuous (CON) or 1h-pulsed exposure (PUL) to CFZ and in the presence or absence of ruxolitinib. The data for each condition was achieved by reading the number of colonies from two dishes (duplicates for each condition) and presented with error bars (*: *p* value < 0.05 comparing ruxolitinib versus combination treatment). (**C**) The % of apoptotic cells in ruxolitinib, 1h-pulsed CFZ and their combination in MF CD34^+^ cells at 24 h. This experiment was performed in duplicate and showed both CFZ and ruxolitinib (Rx) to increase apoptosis in the MF cells, with the highest effect obtained with drug combination. The concentration of ruxolitinib (RX) was 300 nM for all the experiments in this figure. The *p* value for comparing the two conditions was: control versus Rx, *p* = 0.001; control versus CFZ, *p* = 0.0004; Rx versus CFZ, *p* = 0.028; Rx versus Rx+CFZ, *p* = 0.0001; CFZ versus Rx+CFZ, *p* = 0.0002.

CFZ is rapidly eliminated in vivo, with a half-life of ~1 h. Available data from myeloma patients demonstrate that CFZ reaches a C_max_ of 2890 nM after 30 min infusion of 56 mg/m^2^ dose and then declines to undetectable levels after several hours [29,30]. To simulate in vivo CFZ pharmacokinetics, CD34^+^ cells from three MF patients were treated with 300 nM 24 h continuous ruxolitinib, 300 nM 1h-pulsed CFZ or a combination of both (experiment scheme illustrated in Figure S3). The number of viable cells was assessed after 48 h for MF#3” (MF#3” is from the same patient as MF#3 but taken 3 months later) and 72 h for MF#10, MF#19 and MF#20 (Figure 4A). We found that pulsed exposure to CFZ maintained an anti-proliferative effect on primary MF CD34^+^ cells and MNCs, especially in combination with ruxolitinib. For samples MF#3” and MF#20, ruxolitinib in combination with CFZ 300 nM increased the suppressive effect of CFZ, while by itself, reduction of cell viability or growth at 72 h did not occur (Figure 4A). Assessment of colony formation in CD34^+^ cells from MF#28 treated with CFZ either continuously or by pulsed exposure further supported the enhanced suppression of colony formation when cells were treated with combined CFZ and ruxolitinib (although this was statistically significant for the continuous exposure setting only) (Figure 4B).

To explore the mechanism of CFZ-induced suppression of MF cells, apoptosis was measured in primary MF cells following treatment with ruxolitinib, 1h-pulsed CFZ or a combination of both. Analysis of apoptotic cells by the Annexin V method showed increased apoptosis at 24 h with either ruxolitinib or CFZ, and to a higher degree for the combination (Figure 4C and Figure S4) (*p =* 0.001, *p* = 0.0004 and *p* < 0.0001 for control vs. ruxolitinib, CFZ and combination, respectively; Tukey’s multiple comparison test).

### 3.3. Gene Expression Analysis Shows Downregulation of Proteasome Genes by Ruxolitinib

We performed gene expression analysis of primary MF CD34^+^ cells to identify those most affected by ruxolitinib and to define a list of genes of interest for the enrichment phase [31,32].

**Figure 5:**
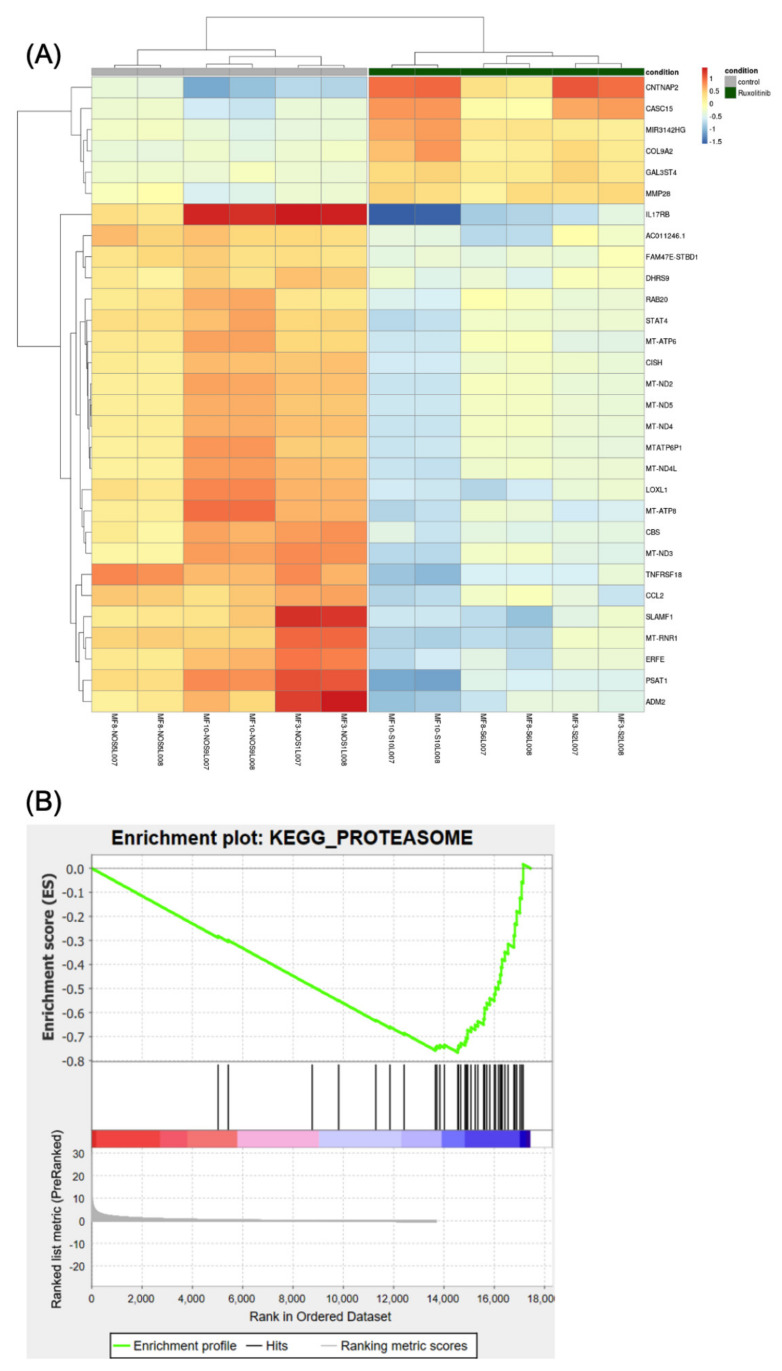
The differential expression analysis. (**A**) Heatmap showing the 30 most statistically relevant genes which are differentially expressed between the ruxolitinib-treated cells and the untreated control. The heatmap is organized into two parts grouping the untreated samples (the first 6 columns on the left side of the heatmap, also indicated by a grey bar at the top of the heatmap) from the treated samples (the remaining 6 columns of the heatmap, also indicated by a dark-green bar at the top). The heatmap provides clustering both on the rows (genes) and between the columns (samples). (**B**) Plot reporting the profile for the Enrichment Score (ES) of the proteasome gene set members. The gene set members are represented as black bars. They are listed in order of ES, that is the genes at the left side are the most upregulated, whereas the ones on the right side are downregulated. The figure shows that most of the dysregulated genes in the proteasome pathway (gene set) are downregulated.

Expression analysis assessed differential expression in 17,435 genes. Using our thresholds for statistical and biological relevance, 186 genes were significantly differentially expressed, of which 46 were upregulated (log_2_ fold change > 1 in absolute value) and 140 were downregulated (log_2_ fold change < 1 in absolute value) in treated cells compared to untreated cells (Table S5). The top 30 genes are shown in Figure 5A. Most of the genes were downregulated by ruxolitinib and the majority of these downregulated genes are involved in the immune–inflammation process. The list of differentially expressed genes to provide as input into the gene set enrichment analysis (GSEA) was ranked using the following metrics: −log_10_ (*p* value) * sign (log fold change) [33]. Among the top 10 downregulated pathways (Table S6), we observed the proteasome family, suggesting ruxolitinib treatment has an inhibitory effect on the gene expression of this pathway. The GSEA showed negative enrichment of the proteasome gene set in ruxolitinib treated CD34^+^ MF cells (Figure 5B). The pathway having the most significant downregulated gene expression included genes involved in oxidative phosphorylation.

## 4. Discussion

The success of imatinib in the treatment of chronic myeloid leukaemia through the targeting of the *BCR*-*ABL1* kinase has encouraged a similar approach for treating other types of leukaemia whose pathogenesis is driven by an activated kinase. Ruxolitinib, a *JAK1*/*JAK2* inhibitor, was developed to suppress *JAK2* kinase activity in a similar way to *ABL1* TKIs targeting *BCR*-*ABL1*. However, despite improving clinical symptoms, ruxolitinib has not significantly reduced the burden of clonal disease suggesting that one or more parallel survival pathways are in operation. These parallel pro-survival mechanisms may either be activated in response to ruxolitinib or are already active and sufficient to sustain leukemic stem cells (LSC) survival despite effective targeting of *JAK2*/*STAT*. There is also a possibility that a pro-survival mechanism is activated by the release of the cytokines from the microenvironment surrounding MF cells as suggested for other types of leukaemia [34,35,36].

Using a pooled-shRNA library screen, a forward genetics approach, members of the proteasomal gene family were identified as the most essential survival genes among the 5000 genes targeted by the shRNAs. Our re-identification of *RAN* among the top 13 essential genes in every experiment and setting supports the report of Yan et al. [37] on the importance of this gene for viability of MPN as well as the power of this technique for independently reproducing previous findings of essential genes (Figure 2, Table S2). In the HEL cell line model, the essential role of the proteasomal gene family for viability was observed regardless of treatment with ruxolitinib or CM exposure. Our previous pooled-shRNA library screen of primary AML cells using the identical library did not show proteasome genes among targets having greatest depletion [21], indicating that proteasome genes are not the most effective targets across all myeloid malignancies. Furthermore, chemical validation demonstrated that relatively low doses of proteasome inhibitors were able to significantly affect HEL cell viability, validating the pooled-shRNA library screen results. The measured bortezomib IC_50_ for HEL cells in this study is comparable to the IC_50_ in multiple myeloma cell lines [38] adding evidence to high reliance of these cells on proteasomes for viability and proliferation.

Proteasome inhibition by CFZ demonstrated in vitro synthetic lethality with ruxolitinib in primary MF cells, further supporting the role of the proteasome in MF pathogenesis. Recent analysis of microarray data from 34 MF patients in comparison to healthy controls [23] and an earlier study [24] have shown upregulation of the proteasome degradation pathway in CD34^+^ cells from patients with MF.

*STAT3* is activated by *JAK* phosphorylation in the *JAK*/*STAT* pathway and upregulates the expression of proteasome genes [39]. Ruxolitinib inhibits the *IL6*/*JAK*/*STAT3* pathway [40,41] and this may explain the reduced transcription of proteasome genes as observed in our study. In mammalian cells oxidative phosphorylation controls the transcription of proteasome genes leading to their upregulation as an adaptive response to prolonged exposure to oxidative stress [42,43,44]. Oxidative phosphorylation signalling was at the top of the downregulated pathways by ruxolitinib in MF CD34^+^ cells and may be another mechanism for downregulation of the proteasomal gene family by ruxolitinib. Observation of eight mitochondrial genes among the top downregulated genes in the heatmap may explain the suppression of the oxidative phosphorylation signalling pathway by ruxolitinib however this association requires further investigation using in vitro and in vivo models. Another possible mechanism through which ruxolitinib leads to a decreased expression of proteasome genes is modulation of the adaptative arm of the unfolded protein response (UPR), which is a complex mechanism elicited in the endoplasmic reticulum (ER) by stress. UPR may result in either an adaptive response to stress to promote cell survival or a pro-apoptotic response [45]. Extensive data demonstrate that upregulation of adaptive and pro-survival branch of UPR is an essential pathogenic mechanism in acute and chronic leukaemias [46]. More recently, UPR has emerged as a potential target in *CALR* and *JAK2*-mutated MPNs [47,48,49]. Upregulation of proteasome gene expression is one of the components of the adaptative UPR [50] and there is evidence showing involvement of the JAK2/STAT3 axis in modulation of the ER stress, leading towards cellular adaptation and survival [51,52].

Proteasome activity is required for NF-κB activity [53,54] and constitutive NF-κB activity has been shown in MF cells [55], which protects them against apoptosis [55,56,57,58]. The apoptotic activity of CFZ in MF cells may be due to its role in suppressing NF-κB as demonstrated for other haematological malignancies [59,60]. In addition to the reduced expression of proteasome genes by ruxolitinib in this study (a finding further supporting the use of this agent in multiple myeloma [61], where the proteasome represents a crucial drug target), further MF cell inhibition was observed by adding CFZ.

We hypothesize that enhanced suppression of MF cells following the addition of CFZ to ruxolitinib is due to the induction of lethal ER stress on the background of ruxolitinib-induced downregulation of proteasome genes and possibly by inhibition of NF-κB activity. The enhanced in vitro inhibition of MF cells by combined CFZ and ruxolitinib was achieved at doses several-fold lower than those observed in patients’ plasma after therapeutic CFZ doses. This suggests that therapeutic CFZ doses may have a stronger inhibitory effect on MF cells compared to our in vitro results.

Pre-clinical results using bortezomib in mouse models demonstrated decreased osteoprotegerin levels and osteosclerosis and increased survival [62], but the findings of the clinical study on MF patients were not encouraging [63,64]. We speculate that the lack of clinical efficacy of bortezomib in MF can be overcome by adding inhibitors of the *JAK2*/*STAT5* pathway, which is the central oncogenic driver of MF and can make cells more vulnerable to the action of the proteasome inhibitor.

We chose to investigate CFZ rather than bortezomib because data from myeloma has shown superior efficacy of CFZ [65]. Furthermore, CFZ is the only available proteasome inhibitor that can induce prolonged inhibition of multiple proteasome subunits within the clinical therapeutic dose range [66]. Unlike bortezomib, CFZ is an irreversible proteasome inhibitor that contains an epoxyketone, which forms a covalent bond with the threonine in the active site of the proteasome [27] causing an irreversible proteasome inhibition.

Furthermore, in order to reach a higher proteasome inhibition and cytotoxic effect, co-inhibition of both β5 and β1 subunits (achieved by bortezomib at 1h-pulsed concentrations of 100–1000 nM) or β5 and β2 subunits (achieved by CFZ at 1h-pulsed concentration of 300–3000 nM) is required; the latter combination is particularly effective against proteasome inhibition–refractory multiple myeloma cell lines or primary multiple myeloma cells. CFZ is the only available proteasome inhibitor able to achieve combined inhibition of the β5 and β2 subunits after intravenous administration [38]. This aspect, along with CFZ’s irreversible binding to the proteasome catalytic domain [25,67] may explain CFZ more potent effect compared to bortezomib.

Finally, we cannot exclude off-target effects of CFZ, such as *PP2A* activation and *MCL1* inhibition [68,69], both of which are important targets in MPN [70,71], and may explain our findings in MF cells.

The effect of ruxolitinib–CFZ combination on proliferation and the colony forming capacity of healthy CD34^+^ cells was not investigated in this study; it is likely that haematological toxicity will represent a major concern of this treatment, in view of the known safety profile of each drug in monotherapy.

In regard to this, CFZ is currently being investigated in combination with ruxolitinib and dexamethasone in the treatment of relapsed/refractory myeloma (https://clinicaltrial.gov, NCT03773107). Once safety data are available and further studies confirm our findings, this drug combination will potentially represent a new platform for assessment in patients with MF.

## 5. Conclusions

In this study, we identified the proteasome family as a potential therapeutic target for MF. Our observations imply that to effectively suppress MF cells, a multitarget therapeutic approach is required.

## Data Availability

The data presented in this study can be made available upon request from the corresponding author.

## Supplementary Materials

The following are available online at https://www.mdpi.com/article/10.3390/cancers13194863/s1, Supplementary Methods: Chemical validation of gene targets on HEL cell line, Chemical validation of gene targets on primary cells, Bioinformatics analysis of RNAseq experiment. Figure S1, The protective role of the microenvironment against ruxolitinib. Figure S2, Correlation between experimental replicates. Figure S3, The 1h-pulsed CFZ in vitro experiment. Figure S4, A. The flowcytometry analysis of the Annexin V/Iodide assay. Table S1, Characteristics of MF patients investigated in this study. Table S2, Top essential genes for viability of HEL cells under various conditions. Table S3, The calculated p value of the comparison between various condition in primary samples treated with ruxolitinib or CFZ. Table S4, The calculated p value of the comparison between various condition in primary samples treated with ruxolitinib, pulsed CFZ and the combination. Table S5, Differentially expressed genes in CD34+ PMF cells treated with ruxolitinib. Table S6, Downregulated signalling pathways by ruxolitinib in CD34^+^ PMF cells.
