# Supplementary material for: Carfilzomib Enhances the Suppressive Effect of Ruxolitinib in Myelofibrosis
Source: Cancers (Basel). 2021 Sep 28;13(19):4863. doi: 10.3390/cancers13194863 (PMC8507927; doi:10.3390/cancers13194863)
Supplement: Supplementary file 1 [file cancers-13-04863-s001.zip › cancers-1381499-supplementary.pdf]

*Supplementary files*

# Carfilzomib Enhances the Suppressive Effect of Ruxolitinib in Myelofibrosis

**Simone Claudiani, Clinton C Mason, Dragana Milojkovic, Andrea Bianchi, Cristina Pelligrini, Antinisca Di Marco, Carme R Fiol, Mark Robinson, Kanagaraju Ponnusamy, Katya Mokretar, Avirup Chowdhury, Michael Albert, Alistair G Reid, Michael W Deininger, Kikkeri N Naresh, Jane F Apperley and Jamshid S Khorashad**

## Supplementary Methods

*Chemical validation of gene targets on HEL cell line*

For MTS assay, 3-5 x $10^3$ HEL cells were seeded in 100 μL of RF10 in each well of a 96-well plate and cultured for 72 h in presence of ruxolitinib 300 nM +/- with the proteasome inhibitor carfilzomib (CFZ) at 10 nM and 50 nM. For proteasome inhibitor $IC_{50}$ determination on HEL cells, MTS assay was performed as described. Cells were exposed to increasing concentrations (0-80 nM) of bortezomib or CFZ for 72 h. Experiments contained 3-5 technical replicates.

*Chemical validation of gene targets on primary cells*

For the 72 h MTS assay, 2-5 x $10^4$ $CD34^+$ cells isolated from healthy donor GCSF-stimulated leukapheresis product and $CD34^+$ cells isolated from peripheral blood from MF patients were suspended in 100 μL of StemSpan (STEMCELL Technologies, Canada) medium supplemented with CC100 in each well of a 96-well plate after 24 h expansion in absence of drug. Different concentrations of CFZ were tested with and without ruxolitinib 300 nM. Experiments were performed in triplicate.

Furthermore, the effect on viability of pulsed-proteasome inhibitor in the presence of ruxolitinib was assessed on both MNCs and $CD34^+$ cells derived from peripheral blood of MF patients.

For MF MNCs, cells were seeded in a 24-well plate at a density of 0.3 x $10^6$/mL in StemSpan and incubated for 1h after the addition of (a) medium with no drug; (b) ruxolitinib 300 nM; (c) CFZ 300 nM; and (d) combination of the two drugs. After 1 h and wash with PBS, cells from the above conditions were resuspended in StemSpam supplemented with CC100 and treated with (a) no drug; (b) ruxolitinib; (c) no drug; and (d) ruxolitinib 300 nM. Trypan blue viability assay was performed after 48 h or 72 h incubation. Results of the trypan blue is presented as the mean of 2-4 counts along with the error bars.

For MF CD34+, cells were exposed for 1 h to CFZ 300 nM +/- ruxolitinib 300 nM (following the same procedure described above for MNCs) (Figure S3). Then, after washing with PBS, two experiments were carried out: 1) 2.5-3 × $10^4$ cells were seeded in each well of a 96-well plate in 100 μL StemSpan + CC100 and treated with medium with (a) no drug; (b) ruxolitinib 300 nM; (c) no drug; and (d) ruxolitinib 300 nM; at 72h MTS was used to assess cell viability; 2) cells were resuspended in StemSpam + CC100 in a 24-well plate (0.3-0.5 x$10^6$ cells/mL) and treated with (a) no drug; (b) ruxolitinib 300 nM; (c) no drug and (d) ruxolitinib 300 nM; trypan blue viability assay was performed after 24 h. Results of the trypan blue is presented as the mean of 2-4 counts along with error bars.

*Bioinformatics analysis of RNAseq experiment*

In order to identify gene regions matching the read sequences, sequencing alignment was performed by using the human genome reference GRCh38.p13 (https://www.ensembl.org/Homo_sapiens/Info/Index).

The alignment was performed with STAR (https://github.com/alexdobin/STAR version '2.7.5a') tool, with the '*--twopassMode Basic*' parameter enabled. The genome index was created from scratch with reference to the GRCh38 genome

(ftp://ftp.ensembl.org/pub/release-100/fasta/homo_sapiens/dna/, primary assembly version). In order to quantify gene expression, we counted the number of reads that map to each gene using ‘featureCounts’ (http://subread.sourceforge.net/ version ‘2.0.1’). The reads were normalised and the differential expression analysis was performed by using the ‘DESeq2’ package (version ‘1.26.0’). Heat maps were produced using the ‘pheatmap’ package on R3.6.0 and included only genes showing adjusted *p* values <0.05 and $\log_2$ fold change > 1 in absolute value.

The Gene Set Enrichment Analysis was performed on all differentially expressed genes (not filtered by thresholds on padj and fold change), by using the ‘GSEA’ software (https://www.gsea-msigdb.org/gsea/index.jsp version ‘4.0.3’) with the ‘*Collapse/Remap to gene symbols*’ parameter enabled in ‘No_Collapse’ mode. We used canonical pathways from KEGG and BIOCARTA as the reference database (version ‘7.1’) in this GSEA analysis. Other parameters were used with default settings: 10 as min number of members, 500 as max number of members, and 1000 as number of permutations.

**Supplementary Figures**

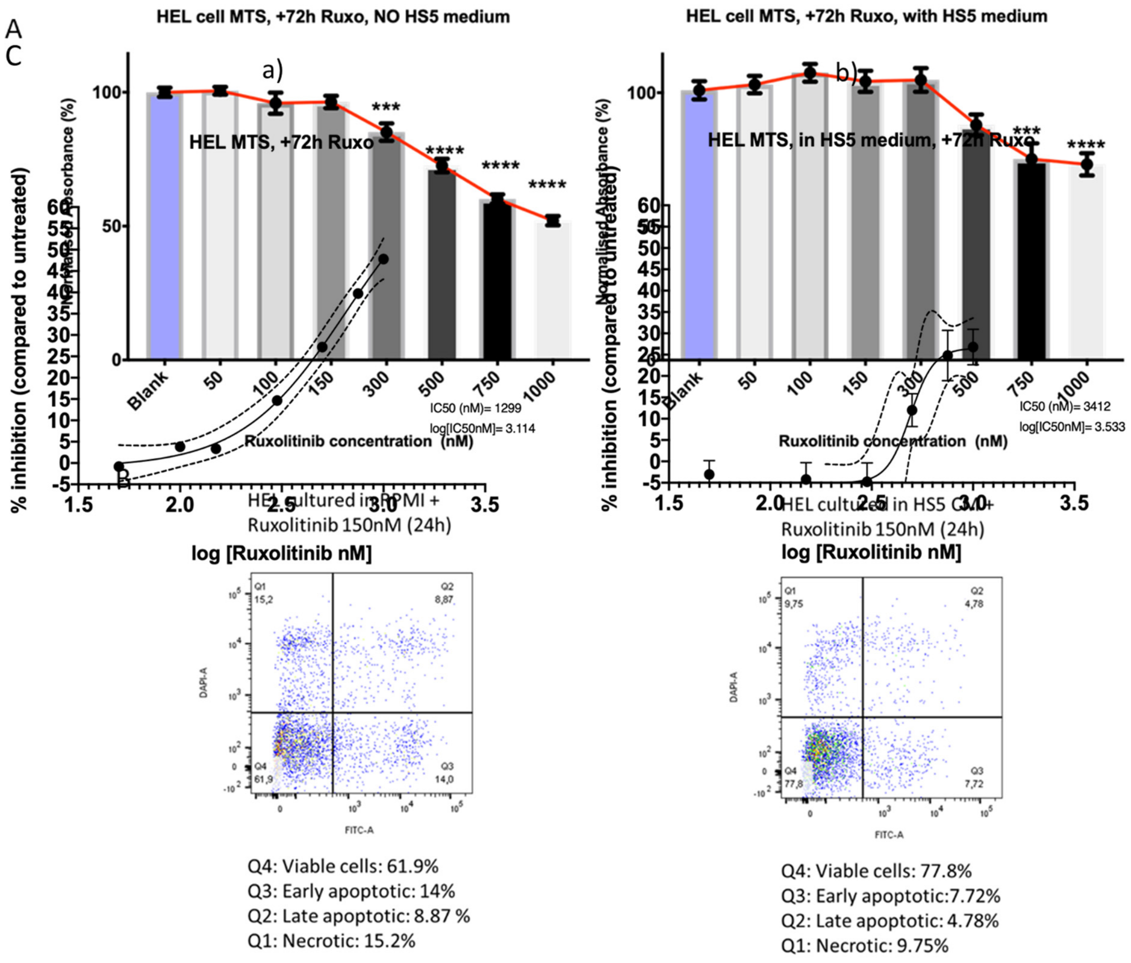


**Figure S1. The protective role of the microenvironment against ruxolitinib.** Figure S1A shows the percent inhibition of proliferation (compared to the untreated control) by increasing concentration of ruxolitinib on HEL cell line cultured in

RF10, derived from 3 representative MTS assays as compared to the same experiment done in the presence of HS-5 medium; each point on the continuous black line represents the mean ± SEM (vertical bars). In the absence of CM there is a continuous reduction in the number of cells which correlated with the increased dose of ruxolitinib at 72h, but CM disrupted this gradual decrease and shifted the inhibitory dose of ruxolitinib to higher level for significant reduction. ****p* < 0.001, **** *p* <0.0001, calculated by Tukey's multiple comparison test. Figure S1B shows the Annexin V-DAPI apoptosis assay on HEL cells treated with ruxolitinib 150nM in RF10 (left) or in presence of HS-5 medium (right); *p* = 0.02, Fisher's exact test. Figure S1C shows that the ruxolitinib $IC_{50}$ of HEL cells cultured in HS-5 medium was nearly three-folds higher compared to culturing only in RF10 medium (3.4 μM versus 1.2 μM, respectively).

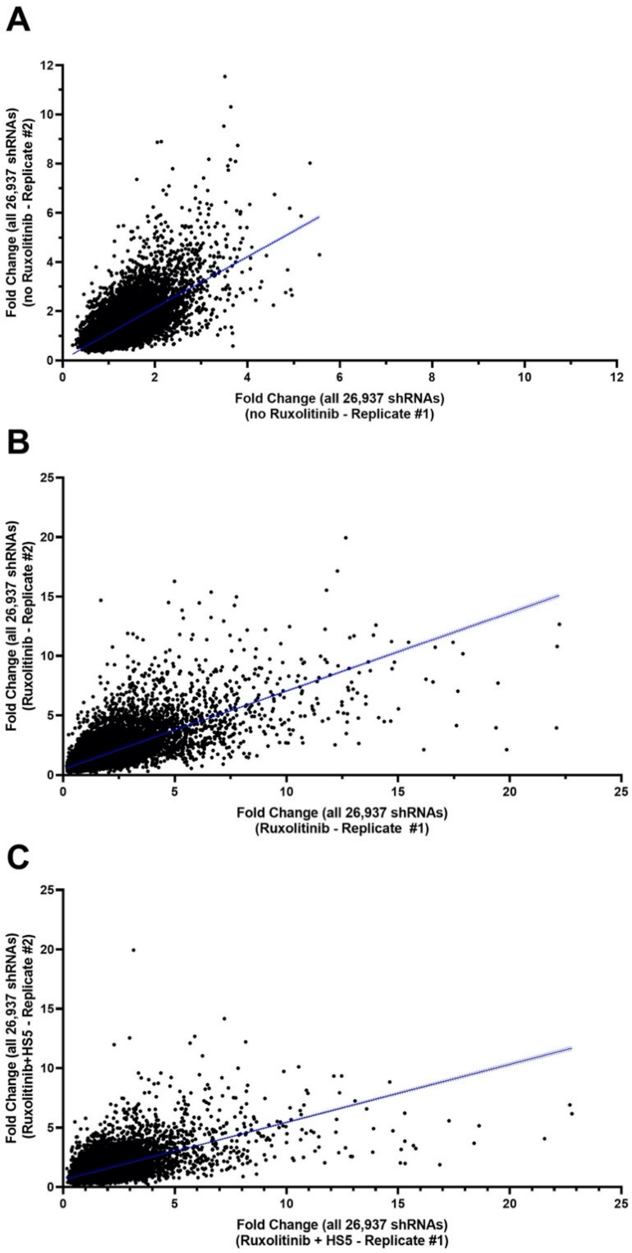


**Figure S2. Correlation between experimental replicates**. The correlation of fold change between experimental replicates is shown for (**A**) no ruxolitinib (setting 1), (**B**) ruxolitnib (setting 2), and (**C**) ruxolitinib plus HS-5 conditioned media. The fold change is calculated as described in the Methods, showing the change in reads present between baseline and at the end of the selection period for each of the 26,937 shRNAs targeting 4,974 genes. The X and Y axes represent the frequency of the normalised shRNA for replicate 1 and 2 for each setting. The Spearman correlations were for setting 1: r=0.635, *p*<0.0001; setting 2: r=0.720, *p*<0.0001; setting 3: r=0.574, *p*<0.0001.

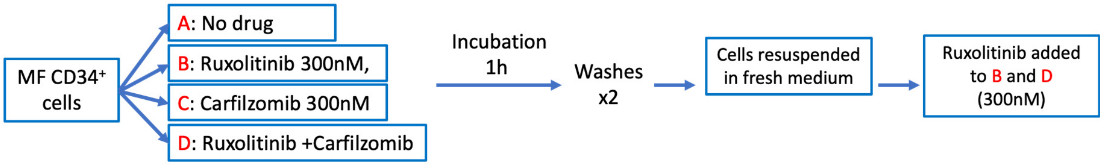


**Figure S3.** The 1h-pulsed CFZ in vitro experiment.The principle of the experiment for mimicking pulsed therapy is shown.

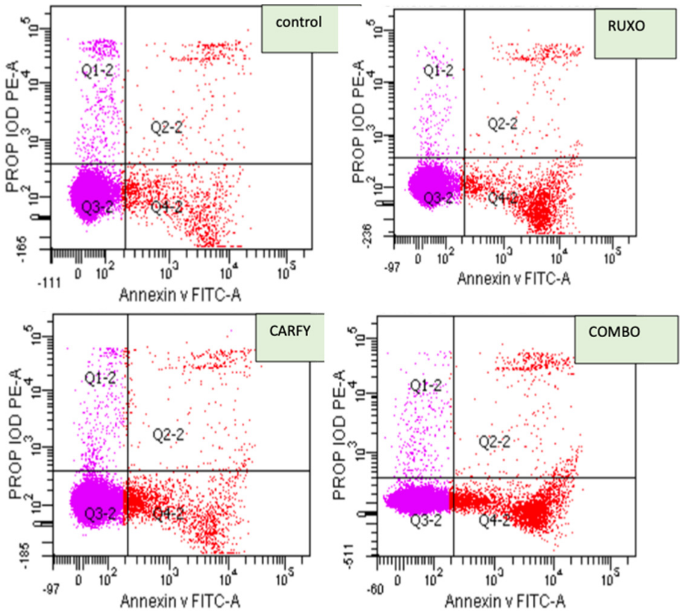


**Figure S4.** A. The flowcytometry analysis of the Annexin V/Iodide assay. The apoptotic cells are highly positive for Annexin which are detected strongly by FITC channel and weakly for the propidium iodide PE-A channel (lower right windows).

**Supplementary Tables**

**Table S1.** Characteristics of MF patients investigated in this study.

| ID | Diagnosis | Sex, Age at sampling | Mutation status (VAF) | Ongoing therapies | WCC (x $10^9$) | Hb (g/L) & Plts (x$10^9$) | Spleen size (cm)/response (c/pr/n) | Symptoms response | Therapy at sample collection? |
|---|---|---|---|---|---|---|---|---|---|
| MF#3 | Primary MF | F, 76 | ***JAK2*** $^{V617F}$ (94%) ***TET2*** (44%) | momelotinib | 23.1 | 90 & 375 | 16.1/n | no | Yes |
| MF#5 | Primary MF | M, 75 | ***JAK2*** $^{V617F}$ (36%) | ruxolitinib (stopped 3 weeks before) | 13.6 | 94 & 34 | NA* | no | No |
| MF#8 | Primary MF | M, 53 | ***MPL*** $^{W515L}$ (74%) ***ASXL1*** (34.4%) | ruxolitinib | 14.6 | 121 & 191 | 28/n | no | Yes |
| MF#9 | Post-ET MF | M, 66 | **Triple-NEG** ***ASXL1*** (36%) | ruxolitinib | 14.6 | 99 & 262 | 20.1/n | no | Yes |
| MF#10 | Primary MF | M, 80 | ***JAK2*** $^{V617F}$(48 %) | ruxolitinib | 23 | 133 & 138 | 17/pr at 5y | yes | Yes |
| MF#14 | Post-PV MF | F, 75 | ***JAK2*** $^{V617F}$(n. d.) | ruxolitinib | 11.7 | 137 & 250 | 19/pr at 6y | no | Yes |
| MF#15 | Primary MF | M, 83 | ***JAK2*** $^{V617F}$(80 %) | ruxolitinib | 15.9 | 109 & 221 | normal/c | no | Yes |
| MF#19 | Primary MF | F, 75 | ***JAK2*** $^{V617F}$(83 %) ***ASXL1*** (10%) | ruxolitinib | 23.2 | 75 & 130 | 23/pr | no | Yes |
| MF#20 | Post-ET MF | F, 60 | ***JAK2*** $^{V617F}$ (94%) | ruxolitinib | 22.3 | 130 & 91 | 30/pr | yes | Yes |
| MF#24 | Primary MF | F, 62 | ***JAK2*** $^{V617F}$ (n.d.) | ruxolitinib | 20.7 | 98 & 179 | 17/pr | no | Yes |
| MF#28 | Post-PV MF | M, 77 | ***JAK2*** $^{V617F}$ (94%) | None | 59.8 | 117 & 278 | 17/NA | NA | No |

VAF = variant allele frequency; WCC = white cell count; Hb = haemoglobin; Plts = platelets; spleen response: c = complete, n = no, pr = progressive splenomegaly. Reported spleen size derives from abdomen ultrasound study. NA = not applicable; nd = not determined. * = patient previously splenectomised. Both spleen response and symptom burden response to treatment are according to the revised IWG-MRT and ELN response criteria (Tefferi A, et al. Blood. 2013;122(8):1395-8).

**Table S2. Top essential genes for viability of HEL cells under various conditions**. Listed are the top 25 essential genes for the viability or proliferation of HEL cells in the presence or absence of ruxolitinib and conditioned medium (CM) as identified by a pooled-shRNA library targeting 4,974 genes for two experimental replicates of each condition.

| | No ruxolitinib (setting 1) | | | | Ruxolitinib (setting 2) | | | | Ruxolitinib + CM (setting 3) | | | |
|---|---|---|---|---|---|---|---|---|---|---|---|---|
| | Experiment 1 | | Experiment 2 | | Experiment 1 | | Experiment 2 | | Experiment 1 | | Experiment 2 | |
| Rank | Gene | Fold Change | Gene | Fold Change | Gene | Fold Change | Gene | Fold Change | Gene | Fold Change | Gene | Fold Change |
| 1 | *CSE1L* | 4.1 | ***PSMD1*** | 8.7 | ***PSMA2*** | 12.7 | *RAN* | 11.7 | *ABCB5* | 11.4 | *RTF1* | 9.2 |
| 2 | *RRM1* | 4 | ***PSMB2*** | 7.7 | *RPS6* | 12 | ***PSMC1*** | 9.7 | ***PSMD3*** | 9 | ***PSMB7*** | 8.6 |
| 3 | *SNRPN* | 3.9 | ***PSMA2*** | 7.4 | *RAN* | 11.3 | ***PSMB2*** | 9.7 | *PRDX2* | 8.4 | ***PSMA2*** | 8.1 |
| 4 | ***PSMA2*** | 3.9 | *SNRPN* | 6.6 | *EIF2S2* | 10.6 | ***PSMA2*** | 9.7 | ***PSMD1*** | 8.3 | *RAN* | 7.6 |
| 5 | *RAN* | 3.7 | *RRM1* | 6.3 | ***PSMC1*** | 9.2 | *RPS13* | 9.6 | *SNRNP200* | 8.2 | *SNRPN* | 7.5 |
| 6 | ***PSMD1*** | 3.6 | *SNRPD2* | 6.2 | *TUBA1C* | 9.1 | ***PSMD11*** | 9.1 | *PHB2* | 8.2 | *RPS13* | 7 |
| 7 | *RTF1* | 3.6 | *HSPE1* | 5.5 | *RUVBL2* | 9 | ***PSMA3*** | 9 | ***PSMA2*** | 8.1 | ***PSMA3*** | 6.9 |
| 8 | ***PSMB2*** | 3.5 | *TUBA1C* | 5.4 | ***PSMB4*** | 9 | *SNRPD2* | 8.9 | *GATA1* | 8 | ***PSMD1*** | 6.5 |
| 9 | ***PSMA3*** | 3.2 | *RPS13* | 5.4 | *SNRPD2* | 8.9 | *RRM1* | 8.6 | *RRM1* | 7.9 | *RRM1* | 6.3 |
| 10 | *EEF2* | 3 | ***PSMA3*** | 5.1 | *RPL14* | 8.8 | *HSPE1* | 8.4 | *RPL11* | 7.9 | *RPL12* | 6.1 |
| 11 | *MPL* | 3 | *SNRNP200* | 4.9 | *RPS11* | 8.6 | *INPP4A* | 8.3 | *TUBA1C* | 7.7 | *RPS3A* | 5.9 |
| 12 | *RPS13* | 3 | ***PSMB7*** | 4.9 | *RTF1* | 8.6 | *RTF1* | 7.9 | *SIN3A* | 7.6 | *SNRPD2* | 5.9 |
| 13 | *SNRPD2* | 2.9 | *RAN* | 4.7 | ***PSMA3*** | 8.6 | *RPL12* | 7.7 | *RAN* | 7.4 | ***PSMB2*** | 5.8 |
| 14 | *EIF2S2* | 2.9 | *EEF2* | 4.6 | ***PSMD1*** | 8.4 | *SNRNP200* | 7.6 | ***PSMB2*** | 7.3 | *RPL10* | 5.7 |
| 15 | *JAK2* | 2.8 | ***PSMB4*** | 4.6 | ***PSMD6*** | 7.9 | *RPL4* | 7.5 | *MPL* | 7.2 | ***PSMC6*** | 5.6 |
| 16 | *RPL12* | 2.8 | *BCL2L1* | 4.4 | *HSPA9* | 7.9 | *EIF2S2* | 7.4 | *EIF2S2* | 7.2 | *SNRNP200* | 5.5 |
| 17 | *SEM1* | 2.7 | *RPL14* | 4.3 | *RPL12* | 7.7 | *HSPA9* | 7.4 | *RUVBL2* | 7 | *KPNB1* | 5.3 |
| 18 | *NEDD8* | 2.7 | *EIF2S2* | 4.3 | *RPS13* | 7.7 | *MPL* | 7.3 | *BCL2L1* | 6.8 | *TUBA1C* | 5.2 |
| 19 | *PHKB* | 2.7 | *RPL13* | 4.3 | *ACTA2* | 7.6 | *FLI1* | 7.2 | ***PSMA4*** | 6.7 | *RPS11* | 5.2 |
| 20 | *CCNG1* | 2.7 | *RPSA* | 4.3 | *PRPF40A* | 7.6 | *RPS6* | 7.2 | ***PSMB7*** | 6.6 | *RPL8* | 5.1 |
| 21 | *SNRNP200* | 2.7 | *RTF1* | 4.2 | *RPSA* | 7.5 | *PRPF40A* | 7 | *STX1A* | 6.4 | *RPL11* | 5 |
| 22 | *CDC40* | 2.7 | *KPNB1* | 4.1 | *PHB2* | 7.3 | *OGDH* | 7 | *INPP4A* | 6.4 | *RUVBL2* | 5 |
| 23 | *FLI1* | 2.7 | ***PSMD6*** | 4.1 | *SNRNP200* | 7.3 | *JAK2* | 6.9 | *DDX23* | 6.2 | *PRDX2* | 4.7 |
| 24 | *PAFAH1B1* | 2.7 | *PHB2* | 4.1 | *EIF1AX* | 7.3 | ***PSMB7*** | 6.9 | *RPS13* | 6.2 | ***PSMA7*** | 4.7 |
| 25 | *RPS11* | 2.7 | *CSE1L* | 4 | *AQR* | 7.2 | *PDHX* | 6.8 | *CD247* | 6 | ***PSMC3*** | 4.7 |

Fold change represents the change in reads observed between baseline and follow-up, with a fold change >1 indicating a depletion from baseline in observed cells having the incorporated shRNA for each gene, as explained in the Methods. A total of 33 proteasomal genes were among the 4,974 targeted in the shRNA library. Additional observations of proteasomal gene family members beyond the top 25 but within the top 1% for each setting and experimental replicate were: experiment 1, setting 1: *PSMD3*, *PSMD7*, and *PSMB3*; experiment 2, setting 1: *PSMC6*, *PSMD2*; experiment 1, setting 2: *PSMB7*, *PSMA4*, *PSMB2*, *PSMD11*; experiment 2, setting 2: *PSMD6*, *PSMD2*, *PSMD1*, *PSMC6*; experiment 1, setting 3: *PSMA3*, *PSMB1*; experiment 2, setting 3: *PSMD2*, *PSMB4*, *PSMD11*, *PSMD6*, *PSMB1*. *JAK2* and *MPL*, which are crucial drivers of MPN pathogenesis, ranked within the top 1.25% of fold change depletions for at least one of the experiments for all settings. Two genes which were in the top 1% of both experiments for setting 3 were not in the top 1% of any experiments for settings 1 and 2: *PSMB1*, *SIN3A*; similarly, unique to setting 2 was only one gene: *AQR*; and similarly unique to setting 1 were two genes: *GTF2F1*, *RACK1*.

**Table S3.** The calculated *p* value of the comparison between various condition in primary samples treated with ruxolitinib or CFZ. The *p* value of comparing any two conditions in these experiments was calculated by unpaired t-test analysis using GraphPad v.9.

| Sample ID | *p* value | | | | | |
|---|---|---|---|---|---|---|
| | Control v Ruxolitinib | Control v CFZ 10 | Control v CFZ 50 | Ruxolitinib v CFZ 10 | Ruxolitinib v CFZ 50 | CFZ 10 v CFZ 50 |
| MF#3 | < 0.0001 | < 0.0001 | < 0.0001 | < 0.0003 | < 0.0001 | < 0.0001 |
| MF#10 | < 0.0001 | < 0.0001 | < 0.0001 | < 0.0001 | < 0.0001 | < 0.0001 |
| MF#15 | < 0.0001 | 0.004 | < 0.0001 | < 0.0001 | 0.07 | < 0.0001 |

**Table S4.** The calculated *p* value of the comparison between various condition in primary samples treated with ruxolitinib, pulsed CFZ and the combination. The *p* value of comparing any two conditions in these experiments was calculated by unpaired t-test analysis using GraphPad v.9.

| Sample ID | *p* value | | | | |
|---|---|---|---|---|---|
| | Control v Ruxolitinib | Control v CFZ 300 | Ruxolitinib v CFZ 300 | Ruxolitinib v Ruxolitinib + CFZ 300 | CFZ 300 v Ruxolitinib + CFZ 300 |
| MF#3 | 0.8 | 0.2 | 0.2 | 0.04 | 0.1 |
| MF#20 | 0.7 | 0.0009 | 0.02 | <0.0001 | 0.001 |
| MF#19 | 0.02 | 0.049 | 0.03 | 0.01 | 0.001 |
| MF#10 | 0.004 | 0.004 | 0.1 | 0.002 | 0.009 |

**Table S5.** Differentially expressed genes in $CD34^+$ MF cells treated with ruxolitinib. This table lists the genes from the most downregulated to the most upregulated ones having a $\log_2$ fold change >1 in absolute value and an adjusted *p* value <0.05.

| Gene | $Log_2$ Fold Change | Standard Error ($log_2$ fold change) | *p* value | Adjusted *p* value |
|---|---|---|---|---|
| *H19* | -6.745 | 1.296 | 1.93E-07 | 2.07E-05 |
| *PAK3* | -5.975 | 1.449 | 3.71E-05 | 1.41E-03 |
| *IL7R* | -4.402 | 0.82 | 7.82E-08 | 9.53E-06 |
| *STC2* | -3.812 | 0.907 | 2.63E-05 | 1.08E-03 |
| *LRRC77P* | -3.316 | 0.575 | 7.99E-09 | 1.42E-06 |
| *ADM2* | -3.261 | 0.561 | 6.06E-09 | 1.14E-06 |
| *GLI2* | -3.123 | 0.631 | 7.32E-07 | 6.35E-05 |
| *IL17RB* | -3.071 | 0.433 | 1.25E-12 | 6.40E-10 |
| *KCNJ15* | -3.07 | 0.87 | 4.15E-04 | 9.21E-03 |
| *CHAC1* | -2.971 | 0.688 | 1.57E-05 | 7.21E-04 |
| *CBS* | -2.891 | 0.413 | 2.52E-12 | 1.16E-09 |
| *FHAD1* | -2.801 | 0.835 | 7.96E-04 | 1.47E-02 |
| *IGFBP5* | -2.794 | 0.534 | 1.68E-07 | 1.86E-05 |
| *CCL24* | -2.577 | 0.639 | 5.52E-05 | 1.96E-03 |
| *TNFRSF18* | -2.569 | 0.241 | 1.81E-26 | 7.90E-23 |
| *SLAMF1* | -2.568 | 0.424 | 1.42E-09 | 3.31E-07 |
| *HSPA7* | -2.49 | 0.812 | 2.18E-03 | 3.14E-02 |
| *ERFE* | -2.49 | 0.348 | 8.31E-13 | 5.37E-10 |
| *SERPINA1* | -2.477 | 0.562 | 1.05E-05 | 5.25E-04 |
| *IL19* | -2.415 | 0.573 | 2.47E-05 | 1.03E-03 |
| *CCL2* | -2.4 | 0.362 | 3.36E-11 | 1.22E-08 |
| *ZNF804B* | -2.389 | 0.707 | 7.29E-04 | 1.39E-02 |
| *LINC01948* | -2.317 | 0.477 | 1.21E-06 | 9.39E-05 |
| *AL138916.1* | -2.304 | 0.8 | 3.99E-03 | 4.76E-02 |
| *EGR1* | -2.292 | 0.543 | 2.46E-05 | 1.03E-03 |
| *AC109466.1* | -2.231 | 0.522 | 1.93E-05 | 8.50E-04 |

| | | | | |
|---|---|---|---|---|
| *RUBCNL* | -2.186 | 0.603 | 2.86E-04 | 6.96E-03 |
| *CCDC3* | -2.155 | 0.559 | 1.17E-04 | 3.50E-03 |
| *ELFN1* | -2.144 | 0.488 | 1.10E-05 | 5.45E-04 |
| *FPR1* | -2.118 | 0.432 | 9.55E-07 | 7.85E-05 |
| *IL2RA* | -2.086 | 0.458 | 5.17E-06 | 3.00E-04 |
| *PSAT1* | -2.051 | 0.246 | 7.23E-17 | 1.05E-13 |
| *DHH* | -1.981 | 0.686 | 3.88E-03 | 4.66E-02 |
| *GGT5* | -1.964 | 0.424 | 3.57E-06 | 2.22E-04 |
| *FAM47E-STBD1* | -1.931 | 0.288 | 1.87E-11 | 7.10E-09 |
| *HP* | -1.93 | 0.655 | 3.23E-03 | 4.12E-02 |
| *LINC00032* | -1.926 | 0.552 | 4.82E-04 | 1.02E-02 |
| *CHDH* | -1.883 | 0.475 | 7.32E-05 | 2.45E-03 |
| *ENPP2* | -1.863 | 0.495 | 1.69E-04 | 4.66E-03 |
| *PPFIA4* | -1.851 | 0.376 | 8.47E-07 | 7.16E-05 |
| *CCDC170* | -1.842 | 0.356 | 2.34E-07 | 2.37E-05 |
| *GLP1R* | -1.836 | 0.604 | 2.37E-03 | 3.33E-02 |
| *IGFBP4* | -1.818 | 0.626 | 3.68E-03 | 4.48E-02 |
| *IER3* | -1.809 | 0.333 | 5.79E-08 | 7.48E-06 |
| *SIGLEC12* | -1.808 | 0.422 | 1.82E-05 | 8.18E-04 |
| *CD80* | -1.796 | 0.418 | 1.77E-05 | 8.00E-04 |
| *FPR2* | -1.786 | 0.37 | 1.40E-06 | 1.05E-04 |
| *CD163* | -1.783 | 0.591 | 2.54E-03 | 3.49E-02 |
| *IL2RB* | -1.774 | 0.567 | 1.77E-03 | 2.68E-02 |
| *SOCS3* | -1.738 | 0.423 | 3.93E-05 | 1.48E-03 |
| *TGFBI* | -1.725 | 0.413 | 2.98E-05 | 1.18E-03 |
| *SLC9A2* | -1.711 | 0.479 | 3.58E-04 | 8.24E-03 |
| *HORMAD2* | -1.695 | 0.44 | 1.17E-04 | 3.51E-03 |
| *OSM* | -1.692 | 0.457 | 2.11E-04 | 5.54E-03 |
| *AF127936.1* | -1.69 | 0.512 | 9.69E-04 | 1.71E-02 |
| *MT-RNR1* | -1.688 | 0.216 | 5.48E-15 | 4.78E-12 |
| *LOXL1* | -1.665 | 0.187 | 4.91E-19 | 1.43E-15 |
| *SERTM2* | -1.663 | 0.526 | 1.57E-03 | 2.46E-02 |
| *LINC02204* | -1.646 | 0.393 | 2.82E-05 | 1.14E-03 |
| *CFH* | -1.645 | 0.3 | 4.30E-08 | 5.73E-06 |
| *AL109837.3* | -1.641 | 0.297 | 3.17E-08 | 4.50E-06 |
| *AL031293.1* | -1.625 | 0.364 | 8.31E-06 | 4.41E-04 |
| *ULBP1* | -1.623 | 0.487 | 8.61E-04 | 1.56E-02 |
| *PIM1* | -1.616 | 0.381 | 2.25E-05 | 9.67E-04 |
| *AC015911.11* | -1.612 | 0.497 | 1.18E-03 | 1.99E-02 |
| *RNVU1-31* | -1.609 | 0.393 | 4.18E-05 | 1.55E-03 |
| *BATF3* | -1.604 | 0.32 | 5.20E-07 | 4.72E-05 |
| *CACNA2D1* | -1.596 | 0.394 | 5.21E-05 | 1.86E-03 |
| *MMP9* | -1.583 | 0.273 | 6.64E-09 | 1.22E-06 |
| *ADGRG3* | -1.582 | 0.427 | 2.08E-04 | 5.47E-03 |
| *IL18RAP* | -1.578 | 0.377 | 2.78E-05 | 1.13E-03 |
| *ST6GALNAC3* | -1.569 | 0.394 | 6.95E-05 | 2.35E-03 |
| *MT-ATP8* | -1.553 | 0.217 | 8.88E-13 | 5.53E-10 |
| *BCYRN1* | -1.534 | 0.529 | 3.70E-03 | 4.50E-02 |
| *TNFRSF8* | -1.532 | 0.378 | 5.01E-05 | 1.81E-03 |
| *GSG1L* | -1.529 | 0.396 | 1.14E-04 | 3.44E-03 |
| *CLEC7A* | -1.527 | 0.482 | 1.53E-03 | 2.42E-02 |

| | | | | |
|---|---|---|---|---|
| *CD22* | -1.516 | 0.422 | 3.27E-04 | 7.75E-03 |
| *MUC1* | -1.485 | 0.266 | 2.25E-08 | 3.42E-06 |
| *SOCS1* | -1.477 | 0.353 | 2.85E-05 | 1.15E-03 |
| *SCN2A* | -1.47 | 0.373 | 8.18E-05 | 2.67E-03 |
| *TREM1* | -1.469 | 0.472 | 1.87E-03 | 2.80E-02 |
| *LIF* | -1.461 | 0.419 | 4.96E-04 | 1.04E-02 |
| *AZIN2* | -1.456 | 0.27 | 6.85E-08 | 8.66E-06 |
| *MT-TF* | -1.454 | 0.326 | 8.43E-06 | 4.44E-04 |
| *ENPP6* | -1.453 | 0.437 | 8.75E-04 | 1.58E-02 |
| *NDUFA4L2* | -1.448 | 0.421 | 5.74E-04 | 1.16E-02 |
| *LTB* | -1.447 | 0.356 | 4.83E-05 | 1.75E-03 |
| *ADAMTS14* | -1.412 | 0.383 | 2.30E-04 | 5.89E-03 |
| *AL034397.2* | -1.411 | 0.333 | 2.26E-05 | 9.67E-04 |
| *ALDH1L2* | -1.394 | 0.264 | 1.30E-07 | 1.51E-05 |
| *RPL41P1* | -1.392 | 0.412 | 7.29E-04 | 1.39E-02 |
| *MT-ND3* | -1.385 | 0.226 | 9.37E-10 | 2.24E-07 |
| *SYN1* | -1.375 | 0.476 | 3.85E-03 | 4.63E-02 |
| *AC005324.3* | -1.365 | 0.351 | 1.01E-04 | 3.16E-03 |
| *SLAMF8* | -1.359 | 0.286 | 2.04E-06 | 1.41E-04 |
| *MT-TL1* | -1.288 | 0.269 | 1.73E-06 | 1.24E-04 |
| *TNFRSF4* | -1.277 | 0.327 | 9.56E-05 | 3.04E-03 |
| *TMEM273* | -1.276 | 0.346 | 2.26E-04 | 5.84E-03 |
| *AC011246.1* | -1.26 | 0.177 | 1.05E-12 | 5.73E-10 |
| *STAT4* | -1.259 | 0.157 | 8.87E-16 | 1.03E-12 |
| *TM4SF1* | -1.257 | 0.336 | 1.82E-04 | 4.96E-03 |
| *GDF15* | -1.249 | 0.3 | 3.15E-05 | 1.24E-03 |
| *AL591506.1* | -1.245 | 0.419 | 2.97E-03 | 3.89E-02 |
| *CHRM3-AS2* | -1.244 | 0.321 | 1.04E-04 | 3.22E-03 |
| *SPP1* | -1.243 | 0.218 | 1.22E-08 | 2.02E-06 |
| *MTATP6P1* | -1.226 | 0.181 | 1.42E-11 | 5.62E-09 |
| *MT-ND4L* | -1.225 | 0.186 | 5.13E-11 | 1.69E-08 |
| *LINC01091* | -1.213 | 0.267 | 5.44E-06 | 3.09E-04 |
| *ID1* | -1.203 | 0.364 | 9.52E-04 | 1.69E-02 |
| *FCRLB* | -1.201 | 0.252 | 1.85E-06 | 1.31E-04 |
| *AL109615.3* | -1.187 | 0.332 | 3.53E-04 | 8.16E-03 |
| *MT-ND2* | -1.175 | 0.178 | 3.91E-11 | 1.39E-08 |
| *MT-ATP6* | -1.171 | 0.17 | 5.34E-12 | 2.22E-09 |
| *CIB3* | -1.17 | 0.397 | 3.17E-03 | 4.08E-02 |
| *CD69* | -1.154 | 0.383 | 2.55E-03 | 3.49E-02 |
| *RN7SL471P* | -1.153 | 0.204 | 1.50E-08 | 2.40E-06 |
| *ICAM1* | -1.151 | 0.377 | 2.24E-03 | 3.20E-02 |
| *MT-ND4* | -1.151 | 0.166 | 4.12E-12 | 1.75E-09 |
| *MT-ND5* | -1.123 | 0.162 | 3.80E-12 | 1.66E-09 |
| *ENOX1* | -1.118 | 0.33 | 7.09E-04 | 1.36E-02 |
| *CISH* | -1.117 | 0.148 | 3.64E-14 | 2.76E-11 |
| *F5* | -1.108 | 0.38 | 3.54E-03 | 4.37E-02 |
| *DHRS9* | -1.108 | 0.173 | 1.48E-10 | 4.08E-08 |
| *MT-ND6* | -1.108 | 0.213 | 1.86E-07 | 2.02E-05 |
| *TRDC* | -1.086 | 0.353 | 2.11E-03 | 3.06E-02 |
| *PPM1E* | -1.066 | 0.349 | 2.27E-03 | 3.23E-02 |
| *MB21D2* | -1.065 | 0.254 | 2.81E-05 | 1.14E-03 |

| | | | | |
|---|---|---|---|---|
| *DDN* | -1.057 | 0.319 | 9.30E-04 | 1.66E-02 |
| *AC084082.1* | -1.054 | 0.311 | 7.02E-04 | 1.35E-02 |
| *ASIC1* | -1.048 | 0.265 | 7.62E-05 | 2.52E-03 |
| *RNY1* | -1.046 | 0.181 | 7.24E-09 | 1.30E-06 |
| *BATF2* | -1.039 | 0.296 | 4.59E-04 | 9.86E-03 |
| *RAB20* | -1.038 | 0.176 | 3.94E-09 | 8.08E-07 |
| *GPR84* | -1.034 | 0.353 | 3.35E-03 | 4.22E-02 |
| *CTSL* | -1.03 | 0.214 | 1.53E-06 | 1.11E-04 |
| *MT-TM* | -1.023 | 0.229 | 8.23E-06 | 4.39E-04 |
| *AC234582.1* | -1.019 | 0.225 | 5.87E-06 | 3.30E-04 |
| *HRH2* | -1.011 | 0.345 | 3.41E-03 | 4.27E-02 |
| *ARG2* | -1 | 0.293 | 6.48E-04 | 1.27E-02 |
| *AP002992.1* | 1.012 | 0.305 | 9.20E-04 | 1.65E-02 |
| *FLT3* | 1.015 | 0.316 | 1.33E-03 | 2.17E-02 |
| *KANK4* | 1.018 | 0.302 | 7.48E-04 | 1.41E-02 |
| *AC092979.2* | 1.041 | 0.33 | 1.60E-03 | 2.48E-02 |
| *MIR3142HG* | 1.059 | 0.16 | 4.06E-11 | 1.42E-08 |
| *AL138963.4* | 1.059 | 0.245 | 1.49E-05 | 6.93E-04 |
| *MMP28* | 1.064 | 0.179 | 2.64E-09 | 5.89E-07 |
| *TRIM73* | 1.075 | 0.243 | 1.00E-05 | 5.07E-04 |
| *COL9A2* | 1.082 | 0.168 | 1.21E-10 | 3.45E-08 |
| *TOB1-AS1* | 1.082 | 0.344 | 1.65E-03 | 2.53E-02 |
| *LGI4* | 1.088 | 0.299 | 2.72E-04 | 6.70E-03 |
| *AC083870.1* | 1.094 | 0.373 | 3.37E-03 | 4.24E-02 |
| *TMPRSS9* | 1.095 | 0.243 | 6.37E-06 | 3.53E-04 |
| *AP000695.2* | 1.104 | 0.275 | 5.97E-05 | 2.07E-03 |
| *LINC00920* | 1.106 | 0.384 | 4.01E-03 | 4.77E-02 |
| *CRHBP* | 1.16 | 0.237 | 9.87E-07 | 7.94E-05 |
| *AP002907.1* | 1.165 | 0.388 | 2.67E-03 | 3.61E-02 |
| *USP30-AS1* | 1.177 | 0.353 | 8.66E-04 | 1.57E-02 |
| *AC234782.4* | 1.243 | 0.266 | 2.89E-06 | 1.87E-04 |
| *PDZD7* | 1.249 | 0.394 | 1.50E-03 | 2.37E-02 |
| *NTN5* | 1.254 | 0.372 | 7.47E-04 | 1.41E-02 |
| *FSIP2-AS1* | 1.261 | 0.368 | 6.03E-04 | 1.21E-02 |
| *LTK* | 1.293 | 0.265 | 1.06E-06 | 8.40E-05 |
| *TNK2-AS1* | 1.297 | 0.405 | 1.37E-03 | 2.23E-02 |
| *CASC15* | 1.303 | 0.219 | 2.53E-09 | 5.73E-07 |
| *AF165147.1* | 1.324 | 0.366 | 2.99E-04 | 7.22E-03 |
| *LINC02082* | 1.342 | 0.443 | 2.47E-03 | 3.42E-02 |
| *RHBDF1* | 1.353 | 0.465 | 3.59E-03 | 4.40E-02 |
| *GAL3ST4* | 1.36 | 0.209 | 7.79E-11 | 2.41E-08 |
| *AC020656.2* | 1.373 | 0.457 | 2.64E-03 | 3.58E-02 |
| *LINC02718* | 1.383 | 0.322 | 1.73E-05 | 7.86E-04 |
| *AC009560.4* | 1.432 | 0.487 | 3.27E-03 | 4.16E-02 |
| *MEGF10* | 1.457 | 0.492 | 3.03E-03 | 3.96E-02 |
| *LINC02147* | 1.461 | 0.495 | 3.13E-03 | 4.05E-02 |
| *TAS1R3* | 1.462 | 0.37 | 7.72E-05 | 2.54E-03 |
| *ECEL1P1* | 1.485 | 0.46 | 1.24E-03 | 2.06E-02 |
| *LINC02801* | 1.539 | 0.276 | 2.35E-08 | 3.50E-06 |
| *CCDC187* | 1.573 | 0.362 | 1.38E-05 | 6.54E-04 |
| *WSCD2* | 1.612 | 0.546 | 3.16E-03 | 4.07E-02 |

| | | | | |
|---|---|---|---|---|
| *SDK2* | 1.665 | 0.56 | 2.95E-03 | 3.87E-02 |
| *AL136181.1* | 1.82 | 0.534 | 6.63E-04 | 1.30E-02 |
| *AC010745.5* | 1.924 | 0.608 | 1.55E-03 | 2.43E-02 |
| *CNTNAP2* | 2.1 | 0.262 | 9.90E-16 | 1.08E-12 |
| *ADCY2* | 3.22 | 0.833 | 1.10E-04 | 3.38E-03 |
| *MIR3648-2* | 3.665 | 1.255 | 3.48E-03 | 4.33E-02 |
| *PRLR* | 3.71 | 1.087 | 6.39E-04 | 1.26E-02 |

**Table S6. Downregulated signalling pathways by ruxolitinib in CD34+ MF cells.** Listed are all gene sets having a nominal *p* value <0.05. Further descriptions of each column variable are provided at: https://www.gsea-msigdb.org/gsea/doc/GSEAUserGuideTEXT.htm#_Detailed_Enrichment_Results.

| Gene set name (GS) | SIZE | ES | NES | NOM p-val | FDR q-val | FWER p-val | RANK AT MAX | LEADING EDGE |
|---|---|---|---|---|---|---|---|---|
| KEGG_OXIDATIVE_PHOSPHORYLATION | 101 | -0.773 | -2.386 | 0 | 0 | 0 | 2506 | tags=51%, list=14%, signal=60% |
| KEGG_SYSTEMIC_LUPUS_ERYTHEMATOSUS | 96 | -0.744 | -2.298 | 0 | 0 | 0 | 2538 | tags=53%, list=15%, signal=62% |
| KEGG_PARKINSONS_DISEASE | 105 | -0.711 | -2.215 | 0 | 0 | 0 | 1926 | tags=43%, list=11%, signal=48% |
| KEGG_HUNTINGTONS_DISEASE | 147 | -0.666 | -2.16 | 0 | 0 | 0 | 2501 | tags=38%, list=14%, signal=44% |
| KEGG_CYTOKINE_CYTOKINE_RECEPTOR_INTERACTION | 140 | -0.656 | -2.129 | 0 | 0 | 0 | 1169 | tags=18%, list=7%, signal=19% |
| KEGG_SPLICEOSOME | 119 | -0.666 | -2.091 | 0 | 0 | 0 | 2935 | tags=51%, list=17%, signal=61% |
| KEGG_PROTEASOME | 40 | -0.766 | -2.013 | 0 | 0.00015 | 0.001 | 2886 | tags=73%, list=17%, signal=87% |
| KEGG_AMINOACYL_TRNA_BIOSYNTHESIS | 39 | -0.756 | -2.005 | 0 | 0.00013 | 0.001 | 2225 | tags=49%, list=13%, signal=56% |
| KEGG_ALZHEIMERS_DISEASE | 131 | -0.619 | -1.979 | 0 | 0.00061 | 0.005 | 2501 | tags=34%, list=14%, signal=39% |
| KEGG_DNA_REPLICATION | 31 | -0.775 | -1.979 | 0 | 0.00055 | 0.005 | 2688 | tags=71%, list=15%, signal=84% |
| KEGG_CARDIAC_MUSCLE_CONTRACTION | 48 | -0.714 | -1.953 | 0 | 0.00109 | 0.01 | 2475 | tags=44%, list=14%, signal=51% |
| KEGG_GLYCINE_SERINE_AND_THREONINE_METABOLISM | 25 | -0.793 | -1.926 | 0 | 0.00184 | 0.019 | 799 | tags=28%, list=5%, signal=29% |
| BIOCARTA_TH1TH2_PATHWAY | 17 | -0.845 | -1.919 | 0 | 0.00203 | 0.023 | 193 | tags=12%, list=1%, signal=12% |
| KEGG_MISMATCH_REPAIR | 22 | -0.783 | -1.896 | 0 | 0.00278 | 0.033 | 2688 | tags=73%, list=15%, signal=86% |
| KEGG_JAK_STAT_SIGNALING_PATHWAY | 94 | -0.608 | -1.858 | 0 | 0.00559 | 0.072 | 1277 | tags=17%, list=7%, signal=18% |
| KEGG_CITRATE_CYCLE_TCA_CYCLE | 27 | -0.719 | -1.831 | 0 | 0.00793 | 0.104 | 1360 | tags=26%, list=8%, signal=28% |
| KEGG_SELENOAMINO_ACID_METABOLISM | 22 | -0.763 | -1.82 | 0.00358 | 0.00859 | 0.119 | 2152 | tags=36%, list=12%, signal=41% |
| KEGG_FRUCTOSE_AND_MANNOSE_METABOLISM | 28 | -0.717 | -1.811 | 0 | 0.00973 | 0.142 | 3254 | tags=57%, list=19%, signal=70% |
| KEGG_AMINO_SUGAR_AND_NUCLEOTIDE_SUGAR_METABOLISM | 39 | -0.693 | -1.809 | 0.00175 | 0.00951 | 0.147 | 3525 | tags=46%, list=20%, signal=58% |
| KEGG_PYRIMIDINE_METABOLISM | 86 | -0.597 | -1.806 | 0 | 0.00965 | 0.156 | 3415 | tags=47%, list=20%, signal=58% |
| KEGG_PPAR_SIGNALING_PATHWAY | 40 | -0.671 | -1.806 | 0 | 0.00925 | 0.157 | 2224 | tags=35%, list=13%, signal=40% |
| KEGG_CYSTEINE_AND_METHIONINE_METABOLISM | 24 | -0.74 | -1.768 | 0.00173 | 0.01519 | 0.256 | 1904 | tags=33%, list=11%, signal=37% |
| KEGG_PYRUVATE_METABOLISM | 33 | -0.67 | -1.757 | 0 | 0.01719 | 0.3 | 1068 | tags=27%, list=6%, signal=29% |
| KEGG_RNA_POLYMERASE | 28 | -0.694 | -1.746 | 0.00349 | 0.01904 | 0.339 | 1443 | tags=36%, list=8%, signal=39% |
| KEGG_GLYCOLYSIS_GLUCONEOGENESIS | 38 | -0.652 | -1.742 | 0.00333 | 0.01889 | 0.35 | 980 | tags=29%, list=6%, signal=31% |
| KEGG_GLUTATHIONE_METABOLISM | 39 | -0.65 | -1.713 | 0.00348 | 0.02724 | 0.489 | 1708 | tags=28%, list=10%, signal=31% |

| | | | | | | | | |
|---|---|---|---|---|---|---|---|---|
| KEGG_TRYPTOPHAN_METABOLISM | 31 | -0.677 | -1.688 | 0.00175 | 0.03671 | 0.601 | 3560 | tags=32%, list=20%, signal=40% |
| KEGG_GALACTOSE_METABOLISM | 22 | -0.701 | -1.672 | 0.00517 | 0.04256 | 0.657 | 3435 | tags=50%, list=20%, signal=62% |
| BIOCARTA_G2_PATHWAY | 24 | -0.678 | -1.665 | 0.01541 | 0.04512 | 0.698 | 3528 | tags=50%, list=20%, signal=63% |
| KEGG_HOMOLOGOUS_RECOMBINATION | 26 | -0.669 | -1.66 | 0.00694 | 0.04609 | 0.715 | 3149 | tags=62%, list=18%, signal=75% |
| KEGG_NUCLEOTIDE_EXCISION_REPAIR | 41 | -0.617 | -1.645 | 0.00664 | 0.05254 | 0.775 | 2724 | tags=46%, list=16%, signal=55% |
| KEGG_BIOSYNTHESIS_OF_UNSATURATED_FATTY_ACIDS | 21 | -0.678 | -1.642 | 0.0123 | 0.05283 | 0.791 | 3473 | tags=48%, list=20%, signal=59% |
| BIOCARTA_NKT_PATHWAY | 18 | -0.708 | -1.59 | 0.0239 | 0.08493 | 0.915 | 2 | tags=6%, list=0%, signal=6% |
| KEGG_ONE_CARBON_POOL_BY_FOLATE | 15 | -0.725 | -1.573 | 0.0239 | 0.09671 | 0.947 | 857 | tags=33%, list=5%, signal=35% |
| KEGG_ARGININE_AND_PROLINE_METABOLISM | 40 | -0.586 | -1.555 | 0.01868 | 0.11145 | 0.974 | 2056 | tags=35%, list=12%, signal=40% |
| KEGG_DRUG_METABOLISM_OTHER_ENZYMES | 21 | -0.657 | -1.538 | 0.04203 | 0.12427 | 0.981 | 3657 | tags=43%, list=21%, signal=54% |
| KEGG_BLADDER_CANCER | 39 | -0.577 | -1.531 | 0.01498 | 0.12943 | 0.988 | 3528 | tags=38%, list=20%, signal=48% |
| KEGG_HEMATOPOIETIC_CELL_LINEAGE | 55 | -0.538 | -1.522 | 0.01557 | 0.13438 | 0.991 | 456 | tags=9%, list=3%, signal=9% |
| BIOCARTA_VEGF_PATHWAY | 25 | -0.633 | -1.51 | 0.02574 | 0.14453 | 0.993 | 3085 | tags=40%, list=18%, signal=49% |
| KEGG_COMPLEMENT_AND_COAGULATION_CASCADES | 35 | -0.568 | -1.494 | 0.04085 | 0.15907 | 0.996 | 1104 | tags=14%, list=6%, signal=15% |
| KEGG_BASAL_TRANSCRIPTION_FACTORS | 28 | -0.592 | -1.489 | 0.03846 | 0.16166 | 0.996 | 1915 | tags=21%, list=11%, signal=24% |
| KEGG_FC_GAMMA_R_MEDIATED_PHAGOCYTOSIS | 84 | -0.497 | -1.488 | 0.01463 | 0.15863 | 0.996 | 2927 | tags=23%, list=17%, signal=27% |
| KEGG_RNA_DEGRADATION | 55 | -0.527 | -1.484 | 0.02254 | 0.1588 | 0.996 | 3572 | tags=45%, list=20%, signal=57% |
| KEGG_CELL_CYCLE | 117 | -0.464 | -1.479 | 0.0121 | 0.16189 | 0.997 | 3589 | tags=43%, list=21%, signal=53% |
| KEGG_PURINE_METABOLISM | 129 | -0.466 | -1.468 | 0.0104 | 0.17087 | 0.997 | 2747 | tags=34%, list=16%, signal=40% |
| KEGG_N_GLYCAN_BIOSYNTHESIS | 46 | -0.529 | -1.467 | 0.03959 | 0.16828 | 0.997 | 2589 | tags=35%, list=15%, signal=41% |

SIZE = Number of genes in the gene set; ES = Enrichment score; NES = Normalized enrichment score; NOM p-val = Nominal *p* value; FDR q-val = False discovery rate; FWER p-val = Familywise-error rate; RANK AT MAX = The position in the ranked list at which the maximum enrichment score occurred; LEADING EDGE = Three statistics used to define the leading-edge subset.
